# Integrating acoustic, prosodic, and phonological features for automatic Alzheimer’s detection

**DOI:** 10.3389/fnagi.2026.1786269

**Published:** 2026-04-16

**Authors:** M. Zakaria Kurdi

**Affiliations:** 1 Meharry Medical College, Nashville, TN, United States; 2 Vanderbilt Memory and Alzheimer’s Center, Nashville, TN, United States

**Keywords:** Alzheimer’s disease, acoustics, speech biomarkers, phonology, prosody, text & speech mining, neurodegenerative disease

## Abstract

**Introduction:**

Early and accurate diagnosis of Alzheimer’s disease (AD) is critical for effective intervention. While prior research has explored speech-based biomarkers for AD, the role of acoustic, prosodic, and phonological features, individually and in combination, remains underexplored.

**Methods:**

This study systematically investigates a broad set of continuous acoustic and prosodic features alongside discrete phonological features for AD detection. Experiments were conducted using the publicly available ADReSS Challenge dataset to ensure comparability with prior work. We evaluated feature importance using four feature ranking methods and assessed classification performance using seven machine learning models. Both individual feature groups and their combinations were analyzed.

**Results:**

Acoustic features demonstrated strong predictive performance, and the best results were obtained by combining acoustic, prosodic, and phonological features. The optimal configuration, using ensemble learning, achieved an F1-score of 0.89. This performance surpasses previous speech-only approaches on the ADReSS dataset and approaches the best overall reported result. Additional validation on the Delaware corpus (focused on mild cognitive impairment) and a longitudinal speech sample from President Ronald Reagan yielded positive results.

**Discussion:**

These findings indicate that different layers of speech representation provide complementary information for detecting cognitive decline. The results suggest that speech characteristics beyond lexical content are highly informative for AD detection. This highlights the potential of multi-layered, non-invasive, and lexicon-independent speech analysis as a tool for early diagnosi sof Alzheimer’s disease.

## 1 Introduction

Aging is accompanied by gradual cognitive, motor, and neurological changes that influence speech production. Older adults often exhibit reduced fluency, slower articulation, decreased articulatory precision, and prosodic flattening. These speech patterns reflect underlying declines in executive control, motor coordination, and neural efficiency (Linville, 2001). Because these alterations emerge naturally in spontaneous communication, speech offers a rich, unobtrusive window into neurocognitive health.

Alzheimer’s Disease (AD) is a major public health concern that disproportionately affects aging populations. According to the Alzheimer’s Association (2025), more than 7.2 million Americans live with AD. Early detection is critical for enabling timely intervention, which can delay cognitive decline and improve quality of life (Rasmussen and Langerman, 2019). However, existing diagnostic practices, such as neuroimaging, cerebrospinal fluid analysis, and lengthy cognitive assessments, are often invasive, costly, and difficult to scale, creating barriers to early screening and monitoring.

Speech has emerged as a promising, non-invasive digital biomarker for detecting cognitive impairment associated with AD. Subtle alterations in phonology, prosody, and acoustic quality may precede more evident cognitive symptoms, providing an early indicator of disease onset (Cera et al., 2023; Koo et al., 2020; Haider et al., 2019; Szatloczki et al., 2015). Advances in machine learning (ML) have further enabled automated analysis of speech patterns, offering potentially scalable and remote assessment solutions for clinical and telehealth settings (Fernández Montenegro et al., 2020). Nevertheless, many prior approaches have focused on limited features sets that has not been selected based on objective criteria, reducing diagnostic power and generalizability across languages and recording conditions.

In this study, we develop and validate an explainable machine learning framework that integrates a broad spectrum of speech features, including segmental (phonological), acoustic, and prosodic cues, to detect AD. This multidimensional design captures complementary aspects of speech production and is robust to linguistic and recording variability, making it particularly suitable for aging populations with diverse backgrounds.

We trained our model on the ADReSS Challenge dataset, a benchmark for AD speech analysis, and validated it on two independent corpora: the Delaware Mild Cognitive Impairment (MCI) corpus and a longitudinal dataset that we collected from President Ronald Reagan’s public speeches. This dual external validation provides evidence of generalizability across recording conditions, disease stages, and topical contexts.

To enhance clinical interpretability, we applied Explainable Artificial Intelligence (XAI) methods to identify which speech features most strongly influenced model decisions. By making the prediction process transparent, our approach supports clinician trust and facilitates the integration of speech biomarkers into clinical workflows.

From a translational perspective, our long-term goal is a patient-centered digital health tool that enables self-administered speech recordings via smartphones or online platforms for early AD screening and continuous monitoring. Such an approach could reduce diagnostic burden, improve accessibility, and empower older adults to engage proactively in their cognitive health management.

The remainder of this paper is structured as follows: section 2 provides a review of relevant literature and highlights existing gaps in speech-based AD detection. Section 3 outlines the methodology and datasets used in this study. Section 4 explains the feature extraction process and examines how AD affects various speech characteristics. Section 5 presents the results along with model interpretation. Section 6 offers a discussion of the findings, their clinical implications, and the study’s limitations. Finally, section 7 concludes the paper and discusses future directions.

## 2 Related work

Extensive research has examined the impact of AD on language, with a significant focus on lexical features. Early evidence suggests that AD primarily affects lexical retrieval, which can manifest as word-finding difficulties and reduced vocabulary richness (Kurdi, 2024a; Mirheidari et al., 2018; Fraser et al., 2016; Garrard et al., 2005; Hebert et al., 2000; Hier et al., 1985). Clinically, lexical changes serve as early indicators of cognitive decline and have been used to support diagnosis in both research and practice. Other studies focused on the syntax of sentence (Nasiri et al., 2022; Eyigoz et al., 2020). Studies combining lexical and acoustic features have consistently reported improved AD detection, highlighting the potential of multimodal approaches (Cummins et al., 2020).

More recently, speech has emerged as a promising non-invasive biomarker for AD, offering scalable and accessible tools for early screening. Research has progressed along two main directions: some studies investigate theoretical correlations between AD and specific speech characteristics (Saeedi et al., 2024; Xue et al., 2021; Mueller et al., 2018; Ranasinghe et al., 2017; Yancheva et al., 2015; Forbes-McKay et al., 2013), while others focus on automated AI-based detection models (e.g., Kurdi, 2024b; Luz et al., 2020; Jarrold et al., 2014). Both approaches aim to identify clinically relevant speech changes that can detect AD before significant cognitive decline occurs.

Phonetic and phonological speech features have also been explored. Early evidence suggested that phonological and articulatory impairments appear in the earliest stages of AD (Croot et al., 2000), and more recent studies confirm that these deficits persist throughout disease progression (Cera et al., 2023). Features such as pause rate and segment duration have been linked to AD severity (Fernández, 2022; Cho et al., 2022). Clinically, these markers may indicate subtle disruptions in speech motor control and temporal organization, providing complementary information to lexical deficits. Recent cross linguistic work by Pérez-Toro et al. (2025) showed that speech timing features, including pause and speech rate, were significantly impacted by AD in both English and Spanish. Remote speech assessments have also been shown to reliably detect amyloid-beta pathology in cognitively unimpaired individuals, reinforcing the potential of speech as an early, objective biomarker (van den Berg et al., 2024).

Acoustic and prosodic features, such as speech rate, pitch variation, and temporal rhythm, have been extensively investigated. Studies report slower speech, increased pauses, and temporal disorganization in AD patients. These characteristics are observable across tasks like picture description or reading aloud (Hoffmann et al., 2010; López-de-Ipiña et al., 2013; Martínez-Sánchez et al., 2013; Satt et al., 2014). They also correlate with disease severity and can provide quantifiable indicators for clinical monitoring (Parlak et al., 2023).

Recent biomarker-linked speech studies indicate that early Alzheimer’s pathology can manifest as measurable disruptions in vocal control and temporal organization. Young et al. (2024) showed that speech-timing irregularities during memory recall tasks correlate with regional tau PET burden, suggesting that microprosodic and temporal instability may reflect early corticolimbic neurofibrillary degeneration. Complementarily, van den Berg et al. (2024) reported that naturalistic speech features, including pausing behavior, articulatory variability, and reduced fluency, track with cognitive vulnerability in preclinical cohorts and maintain reliability under remote acquisition, supporting their value as potentially scalable digital biomarkers. The high-importance predictors in our model, phonatory instability (jitter and shimmer), temporal disruption, articulatory imprecision, and prosodic flattening, align with these pathology-linked feature classes and map onto degeneration of frontotemporal and temporoparietal speech–language networks.

Recent machine learning approaches highlight the diagnostic power of integrating lexical, syntactic, phonetic, and acoustic features. For example, Gonzalez-Atienza et al. (2021) showed that mild AD is associated with shorter, slower, and more effortful speech, with increased disfluencies and pauses, achieving competitive classification performance. The ADReSS Challenge (Luz et al., 2020) has further established benchmarks for speech-based AD detection, with models leveraging fine-tuned language models (BERT, ERNIE) or combined audio-lexical features achieving accuracy about 89% (Yuan et al., 2020; Edwards et al., 2020; Balagopalan et al., 2020). Ensemble learning approaches integrating tree-based and neural classifiers have also improved robustness and interpretability (Pérez-Toro et al., 2022).

Despite these advances, systematic analyses of phonological and prosodic features remain limited. Most studies prioritize classification accuracy rather than exploring the underlying speech changes caused by AD. To our knowledge, no prior work has comprehensively examined prosodic, acoustic, and phonological speech structures from both empirical and conceptual perspectives. Our study addresses this gap by combining rigorous feature-based classification with in-depth linguistic analysis, aiming to provide clinically interpretable insights and explainable AI tools for early AD detection.

## 3 Materials and methods

Four ranking methods that yielded the highest classification performance were selected: ANOVA, Chi-Square (χ^2^), Information Gain, and ReliefF, along with Minimum Redundancy Maximum Relevance (mRMR). Feature ranking using the first four methods was performed with the Orange^1^ library, while mRMR ranking was carried out using the pymrmr Python library^2^ as part of the process of eliminating partially redundant features.

### 3.1 Objective

The primary objective of this paper is to systematically investigate the discriminatory effect of speech features in differentiating between patients with Alzheimer’s disease (AD) and healthy individuals. This exploration aims to provide a foundation for developing machine learning classifiers capable of performing this task automatically. The work is divided into two main phases: first, an individual analysis of how AD impacts a large number of speech features divided into three subgroups: phonological, acoustic, and prosodic features. Secondly, this study aims to identify the most effective combination of features and input length when necessary to optimize classification performance.

### 3.2 Hypotheses

As outlined in section 2, several previous studies have shown that Alzheimer’s disease (AD) has a significant impact on speech. This leads us to hypothesize that speech in individuals experiencing healthy aging is less affected compared to those with AD. Moreover, based on the speech characteristics explored in earlier research, we expect AD to influence all the speech aspects examined in this study. We anticipate that AD will cause significant changes in prosody, acoustic features, phonetics, and phonology. Given that no comprehensive studies have systematically explored the full impact of AD on speech, it is difficult to predict which specific features will be most affected. Finally, considering AD’s impact on continuous acoustic and prosodic features, we hypothesize that it will also affect higher-level phonological features. Additionally, we propose that there may be a positive complementarity between the three feature subgroups, because they cover different aspects of speech, which could enhance AD classification results.

### 3.3 Datasets

The study uses the dataset provided by the ADDReSS challenge organizers (Luz et al., 2020), which is a subset of the DementiaBank corpus, part of the larger TalkBank project (MacWhinney, 2019). This dataset includes recordings and transcripts collected with the Cookie Theft picture description task, with 54 subjects in the training set and 24 subjects in the test set. The training set consists of 108 narrative samples, while the test set includes 48. Both speech audio files and transcripts were analyzed in this study. The ADDReSS dataset was chosen for its balanced representation of age, gender, and conditions, as described by Luz et al. (2020). Additionally, since the dataset has been used in several previous studies, it enables a comparison of our results with those of earlier research utilizing this dataset.

To evaluate the generalizability of our model beyond the ADReSS Challenge dataset, we conducted external validation using the Delaware Corpus, a recent addition to the DementiaBank repository (Lanzi et al., 2023). This corpus includes spontaneous speech samples from individuals with Mild Cognitive Impairment (MCI) and cognitively healthy controls, collected independently from ADReSS. Participants completed a range of narrative and descriptive tasks, including the *Cookie Theft* picture description, *Cinderella* story retelling, procedural instructions (e.g., “how to make a sandwich”), and personal narratives. Speech recordings are accompanied by transcriptions in CHAT format and metadata including cognitive assessments such as the Montreal Cognitive Assessment (MoCA) and Boston Naming Test. Only manually reviewed transcripts, free of automatic speech recognition (ASR) errors, were used in our evaluation to ensure data integrity. The Delaware dataset offers both linguistic and cognitive diversity and represents a clinically relevant benchmark for external validation. Its independent origin, varied task design, of AD, their discriminative value is influenced by the choice of feature-selection method and is enhanced diagnostic scenarios.

Previous studies (Wang et al., 2020; Berisha et al., 2015) have suggested that President Ronald Reagan showed early signs of Alzheimer’s disease during his second term, well before his official diagnosis in 1994. To examine this, forty 5-min passages were sampled from his public speeches. Twenty, 5-min passages, were taken from speeches delivered between 1964 and 1980, before any presumed symptoms. Twenty other passages were taken from speeches between 1988 and 1990, when signs of impairment were thought to emerge. All passages underwent manual quality inspection to ensure accuracy and consistency. Segments containing multiple speakers, background noise, music, applause, or other non-speech artifacts were excluded from the dataset. The speeches were retrieved from the Reagan Library Archives in MP4 format, converted to WAV using *pydub*, and transcribed with the *speech_recognition* library and the Google Speech-to-Text engine.

The ADDReSS challenge, the Delaware, and the Regan datasets used reflect real-world clinical populations with varying cognitive impairment levels, ensuring that the developed model is applicable across typical clinical scenarios.

**FIGURE 1:**
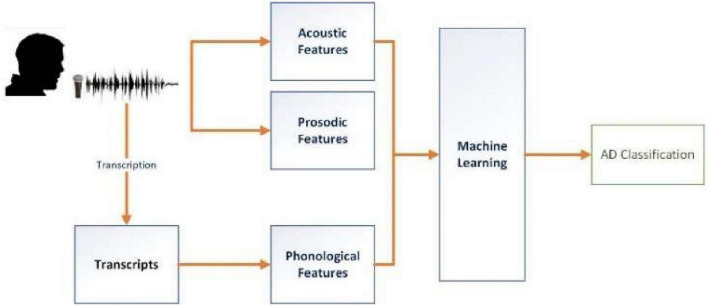
Outline of the work conducted in this paper.

As illustrated in Figure 1, three types of features were extracted: acoustic, prosodic, and phonological. Acoustic and prosodic features were derived directly from the audio wave files. Phonological features were obtained from transcriptions, including those provided by the ADReSS Challenge that were obtained manually and those that we generated via automatic speech recognition (ASR) to assess its impact. These feature sets will be used alone or in combination to train classifiers with different machine learning algorithms.

## 4 Features

Due to the extensive number of features examined in this study, a simplified ranking system was used to facilitate comparison. In this system, a ranking of A indicates that a feature is within the top 10%, B represents the top 25%, C the top 50%, and D the top 75%. A dash (-) signifies that the feature falls outside the top 75%.

To address redundancy, a two-stage feature selection was applied. First, features within each category (e.g., MFCCs) were ranked using mRMR, retaining the top 10% per category. As its names indicate, mRMR picks those features that reduce the redundancy, which is expected in this case given the high similarity between the features of the same type. Second, a global ranking was performed across the selected features to identify the most informative predictors.

### 4.1 Acoustic features

Speech acoustics reflect cognitive and emotional states and are widely used in automatic speech recognition, speaker identification, and detection of mental health and neurodegenerative disorders (Deng et al., 2022; Edwards et al., 2020; Pérez-Toro et al., 2022). Acoustic features were extracted from all speech segments using openSMILE v2.1, a widely used open-source toolkit for automatic speech analysis, particularly in emotion and affective computing (Eyben et al., 2010, 2013), and extensively applied in Alzheimer’s disease research (Luz et al., 2020; Christensen et al., 2020; Pappagari et al., 2020; Edwards et al., 2020). In total, 6,056 acoustic features were generated (Supplementary Table 1).

#### 4.1.1 Audio spectral features

Audio spectral features capture multiple characteristics of speech, including Relative Spectral Transform-Perceptual Linear Prediction (RASTA) and Relative Spectral Filtering. Each characteristic is quantified using standard statistical descriptors such as mean, standard deviation, quartiles, minimum, maximum, skewness, and kurtosis. Audio-based features have previously been applied to Alzheimer’s disease (AD) detection; for example, Shah et al. (2020) used spectral features within a classification framework and reported an accuracy of 60.19. Given the large number of spectral features available, only the top 10 features, based on ranking across the five applied feature-selection methods, are presented.

As shown in Supplementary Table 2, statistical variations of the Rfilter features consistently ranked highest. All listed features appeared within the top 10% in at least three ranking methods, suggesting that these spectral characteristics are strongly influenced by AD.

#### 4.1.2 MFCC features

Mel-Frequency Cepstral Coefficients (MFCCs) represent the short-term power spectrum of speech by applying a cosine transform to the logarithm of the power spectrum scaled on the mel frequency scale. MFCCs are widely used for feature extraction in speech and audio processing, including automatic speech recognition and speaker identification. Given their ability to capture spectral patterns associated with articulatory and phonatory changes, they have been extensively applied in speech-based Alzheimer’s disease (AD) detection. However, prior findings have been mixed. Martinc and Pollak (2020) reported an accuracy of 0.57 using MFCCs with an SVM classifier, whereas Balagopalan et al. (2020) found that MFCCs alone did not significantly distinguish AD from healthy controls. Cummins et al. (2020) used MFCCs within a Bag-of-Audio-Words framework, achieving an accuracy of 0.611 and an F1 score of 0.604.

Kumar et al. (2022) analyzed 442 audio recordings from the DementiaBank dataset using a combination of deep learning and traditional machine learning methods, incorporating speech different types of speech features such as MFCCs. They achieved a best F1 score of 0.86.

In our analysis, the top 10 MFCC features (Supplementary Table 3) were predominantly associated with the Simple Moving Average (SMA). Unlike the top-ranking audio spectral feature (audSpec_Rfilt_sma_de[21]_meanSegLen), none of the MFCC features appeared within the top 10% across all feature-ranking algorithms. This suggests that while MFCCs capture relevant acoustic markers of AD, their discriminative value is influenced by the choice of feature-selection method and is enhanced when combined with complementary acoustic and prosodic features.

#### 4.1.3 Pulse code modulation

Pulse-code modulation (PCM) features capture basic signal energy and spectral properties of speech. Using openSMILE, we extracted three core PCM low-level descriptors: root mean square (RMS) energy, Fast Fourier Transform magnitude (FFT mag), and zero-crossing rate (ZCR). These features have previously shown sensitivity to speech changes in Alzheimer’s disease and related dementias (Kim et al., 2018; Shah et al., 2020; Lal et al., 2024).

After intra-group mRMR ranking, the top 10 PCM features were retained (Supplementary Table 4). Notably, no RMS-based features ranked in the top 10; all selected features were derived from FFT magnitude, indicating that spectral energy distribution carries greater discriminative information than overall signal energy in this older-adult cohort.

#### 4.1.4 Log harmonic to noise and voice final unclipped

The harmonics-to-noise ratio (HNR) quantifies the relative energy of periodic versus aperiodic components in speech, with lower values indicating increased vocal irregularity commonly observed in aging and neurodegenerative conditions (Martínez-Nicolás et al., 2021). The logarithmic version (Log HNR) is used for better numerical stability. Although one study found no significant group differences in HNR between individuals with Alzheimer’s disease (AD) and healthy controls (Parlak et al., 2023), Log HNR-derived features have contributed to successful speech-based AD classification in multiple prior works (Vigo et al., 2022).

We also included voicing-related features from final unclipped speech segments (voicing probability at phrase endings), which influence perceived speech naturalness and have been employed in automatic speech recognition and clinical voice assessment (Catts and Jensen, 1983).

The top six Log HNR and voicing features retained after intra-group ranking are listed in Supplementary Table 5. Supplementary Figure 1 shows that the log HNR and voicing features consistently fall into the lowest subgroup, which is consistent with the low rankings seen in Supplementary Table 5. This can be partly explained by the smaller number of variants in this subgroup compared to others. In contrast, the Audio Spectral Features, represented in blue, consistently achieve the highest rankings across all feature selection methods. This strong performance is likely attributed to the larger number of variants in this subgroup, as well as the higher number of features that made it into the top ranks.

### 4.2 Prosodic features

Cognitive impairments, particularly in memory and executive function, have been linked to altered prosodic patterns, including reduced pitch-related measures and increased jitter, shimmer, and amplitude variability (Martínez-Sánchez et al., 2013; Meilán et al., 2012). Prosody reflects rhythm, melody, and intonation in speech and contributes to meaning, emotion, and emphasis. It can be broadly categorized into prosodic acoustic features and prosodic phonological features. Prosodic acoustic features capture continuous aspects of speech, such as fundamental frequency (F0) variation, speech rate, and pitch; these are examined in this section and summarized in Supplementary Table 6. Prosodic phonological features, which describe discrete elements such as syllables and phoneme segments, are addressed in section 4.3.

A total of 470 prosodic features were extracted. Because jitter and shimmer include a large number of highly redundant measures, a two-stage feature selection process was applied. First, the most informative jitter and shimmer features were identified with mRMR; the top 10 were then combined with the remaining prosodic feature sets.

#### 4.2.1 Fundamental frequency variations

Fundamental frequency (F0), perceived as pitch, is the lowest frequency of a periodic speech signal and reflects the vibration rate of the vocal folds. Variations in F0 capture changes in pitch and intonation over time. This section focuses on overall statistical patterns of F0 variation, including mean, standard deviation, linear regression indicators, and quantile-based measures. In total, 39 F0 variation features were extracted using the openSMILE library.

Previous studies have linked F0 characteristics to cognitive decline, although results remain inconsistent. Parlak et al. (2023) reported a significant negative correlation between Mini-Mental State Examination (MMSE) scores and F0. Meilán et al. (2012) found no association between Alzheimer’s disease (AD) and mean F0, whereas Bae et al. (2023) observed strong correlations between AD and both mean F0 and the 20th percentile of F0. Similarly, Huang et al. (2024) identified significant associations for five of seven F0 measures examined. Given the extensive number of F0 features, only the top 10 ranked measures are presented (Supplementary Table 7), where three variants of the Linear Predictive Coefficient (LPC) appear among the top four. However, partly due to redundancy among these measures, their ReliefF scores were comparatively lower. Other highly ranked F0 descriptors included interquartile ranges, minimum and maximum values, regression-based measures, and rise time.

#### 4.2.2 Pitch

Speech pitch is the perceptual attribute that conveys how high or low a voice sounds and is determined by the frequency of vocal fold vibration. Pitch plays a central role in expressing intonation, emotion, and emphasis in spoken language. Not all speech sounds are voiced, however; for example, the sounds *[s]* and *[p]* are voiceless because they do not involve vocal fold vibration (Kurdi, 2017). Speakers also rapidly adjust their vocal output to compensate for brief perturbations in pitch within their auditory feedback (Ranasinghe et al., 2017). Pitch-related features have been used in several studies to detect Alzheimer’s disease (AD), with promising results reported by Huang et al. (2024), Ambrosini et al. (2019), and Yu et al. (2015). As shown in Supplementary Table 8, two pitch features, meanP and pearModeFirstP, ranked highest across all four feature ranking methods. Other features, such as iqrP, skewP, kurtFisherP, and kurtPearsonP, received high rankings from three methods but a lower ranking with relifF, possibly due to partial redundancies among these features.

#### 4.2.3 Amplitude

Amplitude is a key prosodic parameter reflecting the loudness of speech, defined as the maximum displacement of vibrating particles from their resting position. Prior research shows that individuals with Mild Cognitive Impairment (MCI) exhibit weaker vocal intensity than healthy older adults. Such adults are characterized by increased dysphonia and a higher ratio between the first harmonic amplitude and the F3 amplitude during phonation (Themistocleous et al., 2020). Similar amplitude-related abnormalities have been reported in individuals with Alzheimer’s disease (AD) compared with healthy controls (Tanaka et al., 2017). Amplitude-based features have also been incorporated into recent AD classification studies using acoustic markers (Luz et al., 2020; Hason and Krishnan, 2022). The top 10 ranked amplitude features are summarized in Supplementary Table 9, which also shows greater disagreement among feature-ranking methods for amplitude features than for other acoustic subtypes. For example, mean amplitude received an A ranking with information gain but a D ranking across the remaining methods. Some features, such as the interquartile range (IQR), consistently received A or B rankings, whereas others, including variance, mode, delta, and Pearson Mode Second, ranked consistently low across all ranking approaches.

#### 4.2.4 Intonation

Intonation refers to the melodic contour of speech, reflected through pitch variations over time. Several studies have investigated how Alzheimer’s disease (AD) alters intonational patterns (Mueller et al., 2018). For example, Forbes-McKay et al. (2013) compared melodic contours in approximately 60 participants and found that individuals with mild to moderate AD exhibited reduced intonational variation during both simple and complex description tasks. Intonation features have also been incorporated into AD speech-classification models (König et al., 2015). The top 10 intonation features identified in our analysis are shown in Supplementary Table 10. Across feature-ranking methods, substantial variability was observed. However, the *Ratio peaks* feature consistently ranked among the highest, whereas the *Mean* feature consistently appeared near the bottom across all ranking methods.

#### 4.2.5 Articulation rate and patterns of unfilled pauses

Articulation rate, the speed of producing linguistic units, varies with emotional state, context, speaker characteristics, and language. Slower speech is linked to word-retrieval difficulties (Satt et al., 2014; Pu and Zhang, 2025) and, along with speech tempo and hesitation ratio, distinguishes individuals with Alzheimer’s disease (AD) from healthy controls (Hoffmann et al., 2010; Huang and Yang, 2022). Speech rate is among the most discriminative features for differentiating AD from healthy aging (Martínez-Sánchez et al., 2013), while pause patterns add further diagnostic value (López-de-Ipiña et al., 2013). Changes in speech duration and rhythm may signal early AD (Meilán et al., 2020), and slower speech is also reported in other dementias, including Parkinson’s disease (Skodda and Schlegel, 2008). Recent studies have successfully incorporated speech rate and tempo into AD classification (Huang et al., 2024).

We analyzed three speech-rate features, ratioWords, ratioChars, and ratioSylls, defined as the number of linguistic units (respectively words, characters, and syllables) divided by total utterance duration (Supplementary Table 11). Despite prior evidence linking slower speech to AD, these features consistently ranked low across all four feature-selection methods, with syllables per second performing worst.

Pauses, while partly driven by respiration, also convey linguistic and speaker-specific information (Kurdi, 2024b). AD speech exhibits longer, more frequent, and more variable pauses (Pastoriza-Domínguez et al., 2022; Hason and Krishnan, 2022; Martínez-Nicolás et al., 2021), making silence a widely used marker of cognitive impairment that reflects delays in speech planning and production (Gonzalez-Atienza et al., 2021; Pappagari et al., 2020; Yuan et al., 2020). However, there is no consensus on the minimum pause duration, with cutoffs ranging from 10 ms (Skodda and Schlegel, 2008) to 50 ms (Goberman et al., 2005) or 200 ms (Bunton and Keintz, 2008; Gravelin and Whitfield, 2017).

We computed the ratio of silent segments to total signal duration using thresholds of 10, 25, 50, 100, and 200 ms. Detection was challenged by overlapping speech and background noise. As shown in Supplementary Table 12, none of the silence features ranked highest across methods, though the shortest threshold (10 ms) performed best. No consistent pattern emerged across pause durations, as longer thresholds sometimes ranked higher than shorter ones and vice versa.

#### 4.2.6 Jitter and shimmer

Jitter and shimmer represent micro-prosodic fluctuations in pitch and amplitude, respectively, and are commonly used to assess voice quality and detect vocal pathologies (Wertzner et al., 2005; Teixeira et al., 2013). Recent findings indicate that individuals with Alzheimer’s disease (AD) exhibit significantly higher jitter and shimmer values compared with healthy controls (Parlak et al., 2023). These measures have also been incorporated into automated AD classification studies (Hason and Krishnan, 2022). The top 10 shimmer and jitter features identified in our analysis are presented in Supplementary Tables 13, 14 respectively. As shown in these tables, the rankings of similar features, such as jitterLocal_sma_range and shimmerLocal_sma_qregerrQ, vary considerably across feature-selection methods. At the same time, clear distinctions emerge between the highest- and lowest-ranked jitter and shimmer features, indicating substantial variability in their discriminative value.

#### 4.2.7 Comparison of the prosodic features by types

A comparison of the prosodic features is provided in Supplementary Figure 2. Given that F0 features represent the largest group in the dataset, it is reasonable to expect them to dominate the ranked feature lists. This expectation is largely confirmed: F0 features account for 65.4% of the top features in the ANOVA ranking and 50% in the χ^2^ ranking. Their prominence, however, decreases substantially under information gain (34.6%) and even more under ReliefF (7.7%). This reduction is consistent with the ReliefF algorithm’s tendency to penalize partially redundant features. Amplitude features exhibit the opposite pattern. They play a minimal role in the ANOVA and χ^2^ rankings (3.8 and 7.7%, respectively) but appear more frequently in the information gain and ReliefF lists (11.5 and 26.9%, respectively). Pitch features, by contrast, maintain a relatively strong presence across all four ranking methods. Unfilled pause features do not appear among the top features in the ANOVA results and occupy only a minor share 3.8% in the χ^2^, information gain, and ReliefF rankings.

### 4.3 Phonological features

Phonology examines the formal structure of speech sounds, including segmental elements (e.g., phonemes, syllables) and suprasegmental features such as intonation and stress. Several studies have explored phonological changes in Alzheimer’s disease (AD). Croot et al. (2000) reported early phonological and articulatory impairments in AD, and Cera et al. (2023) found phonological disorders to be the most prevalent across mild, moderate, and severe AD stages compared with healthy controls.

Guided by prior work, including the Index of Phonetic Complexity (IPC) (Akielski, 2022), we analyzed nine features reflecting word-level phonemic structure, including grapheme count (nbLett), phoneme count (nbPhon), and the presence of specific consonant classes (labials, stops, nasals, dorsals, glides, fricatives, affricates, liquids). Features were organized into three subcategories (Supplementary Table 15): consonants, vowels, and supraphonemic features, with consonants further analyzed by manner and place of articulation (Kurdi, 2017).

For each utterance, we computed the ratio of each consonant or vowel type to the total number of phonemes, providing the phonological features used in the analysis.

#### 4.3.1 Consonant features

Consonants are speech sounds produced by obstructing airflow at specific points in the vocal tract, defined as the place of articulation. In this study, we examined standard phonological categories, including bilabial, labiodental, dental, alveolar, post-alveolar, palatal, glottal, lateral, and coronal consonants, analyzing both place and manner of articulation. Consonant Place of Articulation (CPA) was represented by 18 features capturing distinct articulatory locations.

Dental consonants (/θ/,/ð/) ranked highly across all four feature-selection methods (Supplementary Table 16), despite low overall frequency in English (/θ/: 37th of 39;/ð/: 11th). Their prominence in Alzheimer’s disease (AD) speech likely reflects compensatory lexical behavior, as individuals with AD overuse high-frequency function words such as “the,” “this,” and “that,” consistent with prior evidence that AD speakers rely more on closed-class words when semantic retrieval is impaired (Kurdi, 2024a). In contrast, bilabial, coronal, and lateral consonants consistently ranked low, suggesting these phonemes are less informative for distinguishing AD from healthy speech. Mid-ranked palatals showed no clear association with lexical patterns. These results indicate that specific consonant classes, particularly dentals, may serve as indirect markers of lexical-semantic deficits, supporting the hypothesis that AD affects subtle phonological patterns detectable via automated analysis.

Consonant Manner of Articulation (CMA), which reflects how airflow is modified during production, was represented by 27 features. Fricatives and fricative–affricate–liquid (friAffLiq) features consistently ranked highly (Supplementary Table 17). Their prominence cannot be explained solely by frequency, as some fricatives (e.g.,/ʃ/,/ʒ/) are rare, while others (e.g.,/t/,/s/) are common, suggesting an overlap with dental phonemes and reinforcing the relevance of specific consonant classes to AD-related speech changes.

#### 4.3.2 Vowel features

In phonology, vowels are speech sounds produced without significant constriction in the vocal tract, allowing airflow to pass freely and typically forming the nucleus of syllables. Two ratios were computed for each vowel: the proportion of a specific vowel type to the total number of phonemes in a word (e.g., high-to-phon) and the proportion relative to the total number of vowels in the word (e.g., high-to-vow). Eight vowel types were analyzed as features (Supplementary Table 18). Several features, including tense, syllabic, and round vowels, ranked highly across feature-selection methods.

### 4.4 Misc. features

This subset includes syllabic consonants, which can serve as the nucleus of a syllable, and diphthongs, complex vowel sounds that glide from one vowel to another within a single syllable. Supplementary Table 19 presents the ratios of each sound type relative to consonants and to all phonemes, showing variable rankings. Overall, some features, such as syllabic consonants relative to consonants, ranked above average, whereas others, like diphthongs relative to consonants, ranked lower.

### 4.5 Supra-phonemic structure

Supra-phonemic structures are key indicators of phonological complexity. These include general phoneme sequence features, such as consonant clusters (consCluster), defined as two or more consecutive consonants without an intervening vowel, and word-final consonants (endConson). Syllable structure also reflects phonological complexity, comprising an onset (consonants before the nucleus), a nucleus (typically a vowel or diphthong), and an optional coda (consonants after the nucleus). Two linguistic approaches measure syllabic complexity: categorical syllable complexity (Maddieson, 2007) and the sum of maximal onset and coda (Gordon, 2016; Easterday, 2019), which consider the sizes and types of phonemes in these positions. Accordingly, this study uses lenOnset and lenCoda, as well as vowel-type ratios within the syllable nucleus, back vowels (backSyll), front vowels (frontSyll), and diphthongal nuclei (diphSyll), and the average number of syllables per word (avgSyll).

Two features capture suprasegmental structure: primary stress (primStress), marking the strongest syllable, and secondary stress (secondStress), marking strong but less prominent syllables. Phonemic sequences were obtained from the CMU Pronouncing Dictionary; grapheme sequences were used when dictionary entries were unavailable.

As shown in Supplementary Table 20, avgSyll and consCluster rank within the top 10% of features, demonstrating strong discriminative value and supporting the importance of deeper linguistic analysis. Coda consistently ranks higher than Onset, reflecting the optional nature of onsets in English. Stress-related features (primStress, secondStress) consistently rank low, indicating a limited association with Alzheimer’s Disease (AD).

Supplementary Figure 3 illustrates that Consonant Manner of Articulation (CMA) features dominate rankings across all four feature-selection methods. Miscellaneous features consistently rank lowest. Under ReliefF, vowel and Consonant Place of Articulation (CPA) features receive lower rankings, whereas supra-phonemic features achieve relatively higher positions.

## 5 Results and discussion

This section evaluates the predictive power of the speech features described in section 4. The following machine learning (ML) algorithms were applied: Support Vector Machine (SVM), Logistic Regression (LR), Linear Discriminant (LD), Adaptive Boosting (AB) with 100 estimators, Bagging (Breiman, 1994), Random Forest (RF) with maximum depth 2 (Ho, 1995), eXtreme Gradient Boosting (XG) (Chen and Guestrin, 2016) with parameters learning_rate = 0.001, n_estimators = 5,600, max_depth = 5, min_child_weight = 1, gamma = 0, subsample = 0.8, colsample_bytree = 0.9, objective = “binary:logistic,” seed = 25, and a Multilayer Perceptron (MLP) with max_iter = 200, hidden_layer_sizes = 50, activation = “tanh,” solver = “adam,” and alpha = 1e-8. Parameters were selected empirically to optimize performance. These algorithms were chosen based on preliminary experiments comparing them with alternatives such as Decision Trees and Naïve Bayes. All experiments were implemented in Python using Scikit-learn (Pedregosa et al., 2011) and XGBoost. Performance was evaluated using accuracy, recall, precision, and F1-score (see Manning et al., 2008).

Ensemble learning, which combines multiple classifiers, was also explored given its successful application in AD classification (Sarawgi et al., 2020). Three ensemble methods were employed: hard voting, soft voting, and stacking. In stacking, a meta-classifier assigns optimal weights to each base classifier; here, Adaptive Boosting was used to learn these weights from base classifier probabilities. Ensemble results are reported only when they outperform individual classifiers.

Only F1-scores are presented, as other metrics offered no additional insight. F1 was selected both for its widespread use in ML experiments and to align with the ADReSS Challenge, where it is a formally adopted evaluation measure.

### 5.1 Experiments with acoustic features

**FIGURE 2:**
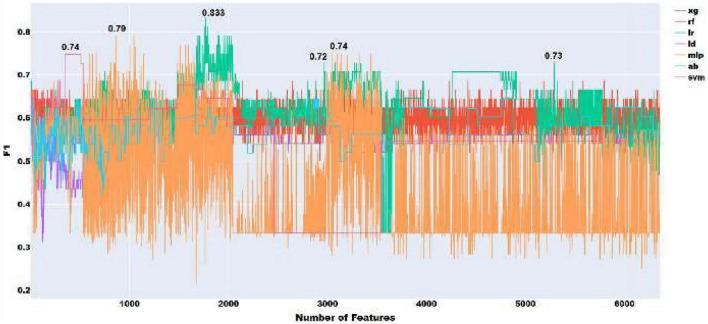
Feature selection experiments with all acoustic features.

To choose the most effective set of acoustic features and evaluate the performance of classifiers using only these features, we adopted the following approach as with the acoustic features. We progressively added features ranked by the four feature ranking methods, starting with the highest-ranked feature and moving eventually to the lowest. The results of this feature selection process are shown in Figure 2, with all the 6376 acoustic features and ANOVA feature selection.

As shown in Figure 2, the highest performance using all acoustic features was an F1 score of 0.83 with logistic regression.

**FIGURE 3:**
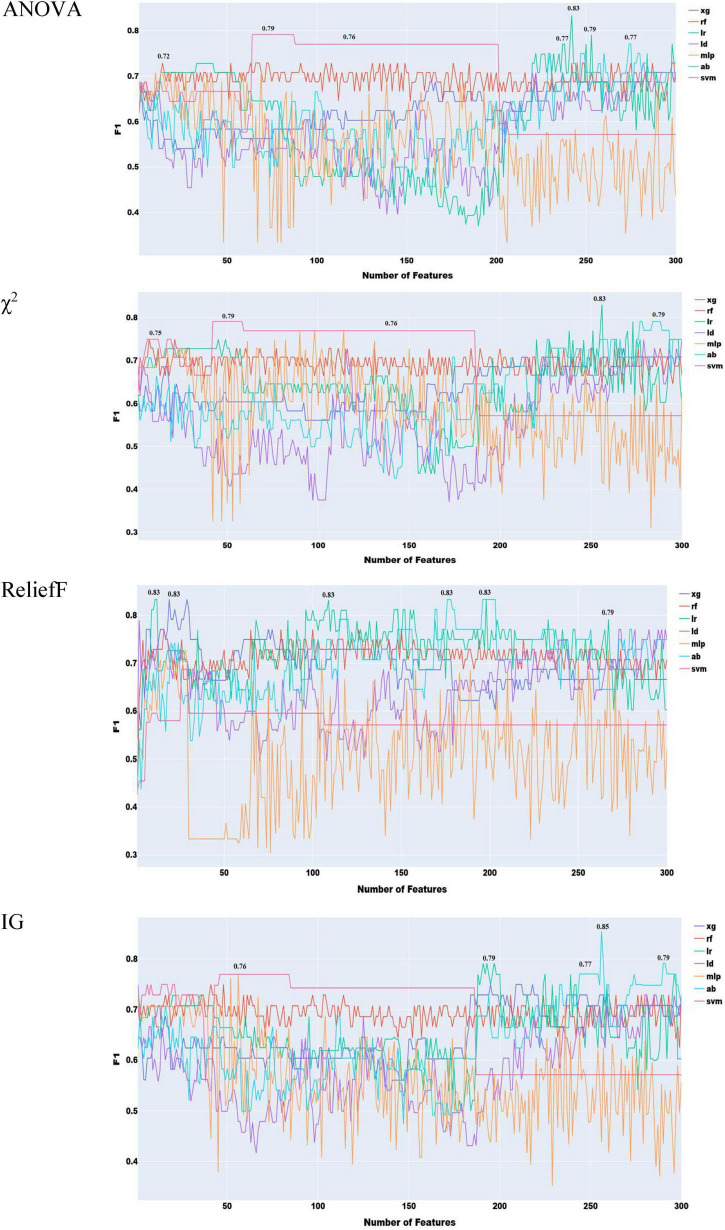
Feature selection experiments with acoustic features only (simple classifiers).

To reduce redundancy in the large feature set, where many features capture different statistical descriptors of the same acoustic properties (e.g., MFCC, F0, jitter), we implemented a two-stage feature selection. First, mRMR was applied to extract the 300 most informative features. Second, this subset was ranked using four additional feature selection methods to identify the most effective combination (Figure 3).

As shown in Figure 3, the highest performance with acoustic features was obtained using the information gain method, yielding an F1 score of 0.85. The remaining feature selection techniques reached their peak performance at an F1 score of 0.83. It is also notable that, with the exception of ReliefF, all methods required a relatively large number of features to achieve optimal results.

To further analyze the generalization behavior of our models, we inspected the confusion matrix of the best-performing classifier, adaptive boosting trained on the top 256 information gain features, which achieved the highest F1 score of 0.85.

As shown in Supplementary Table 21, the classifier has similar rates of true negatives and true positives, suggesting a balanced error pattern. In other words, it performs equally well on both positive AD cases and control cases.

Ensemble learning experiments were conducted. Instead of using all the ML algorithms that we experimented with so far, only those that displayed a steady performance in were retained. Hence, we have used the following ML algorithm in the ensemble experiments: SVM, Random Forest, Adaptive Boosting, Logistic Regression, and XG Boost.

**FIGURE 4:**
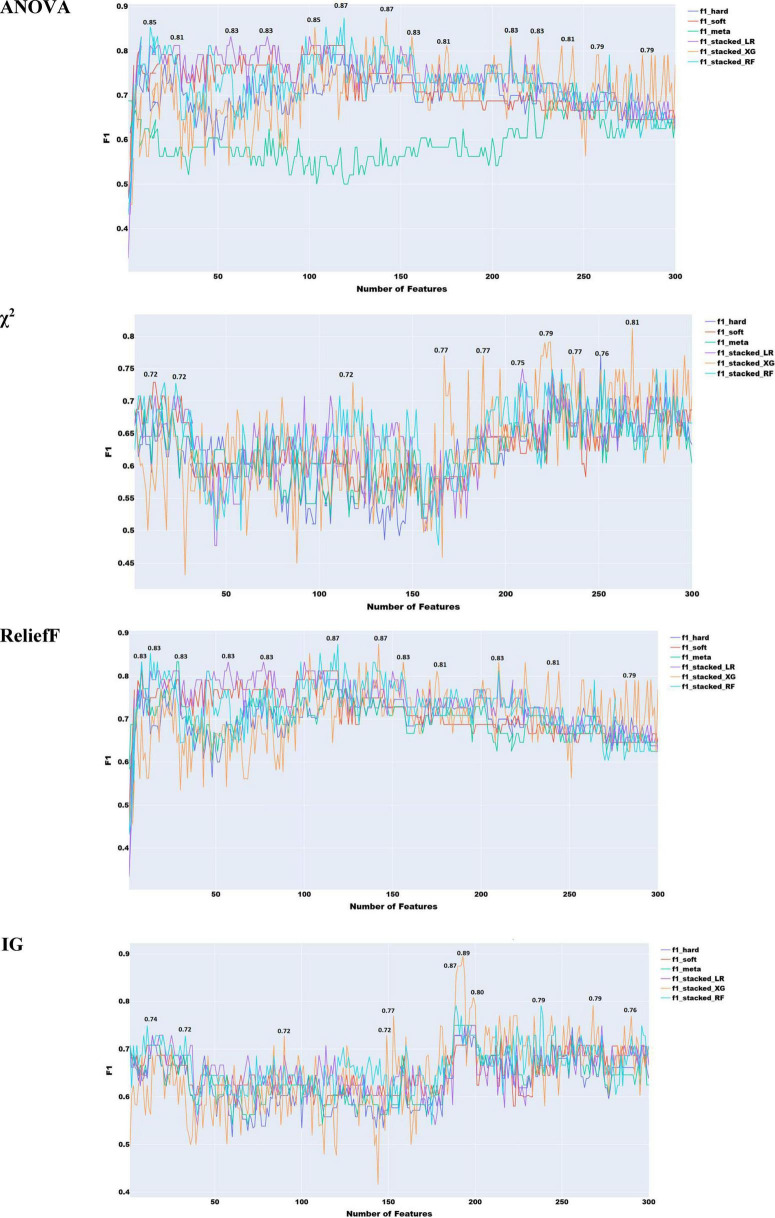
Feature selection experiments with acoustic features only (ensemble learning).

As illustrated in Figure 4, ensemble learning improved the F1 score by 4% when using acoustic features, achieving a score of 0.87 with ReliefF and stacked ensemble approach with both XGBoost and Random Forest. A score of 0.89 was achieved with Information Gain features selection combined with XGBoost with stacked ensemble approach.

### 5.2 Experiments with prosodic features

**FIGURE 5:**
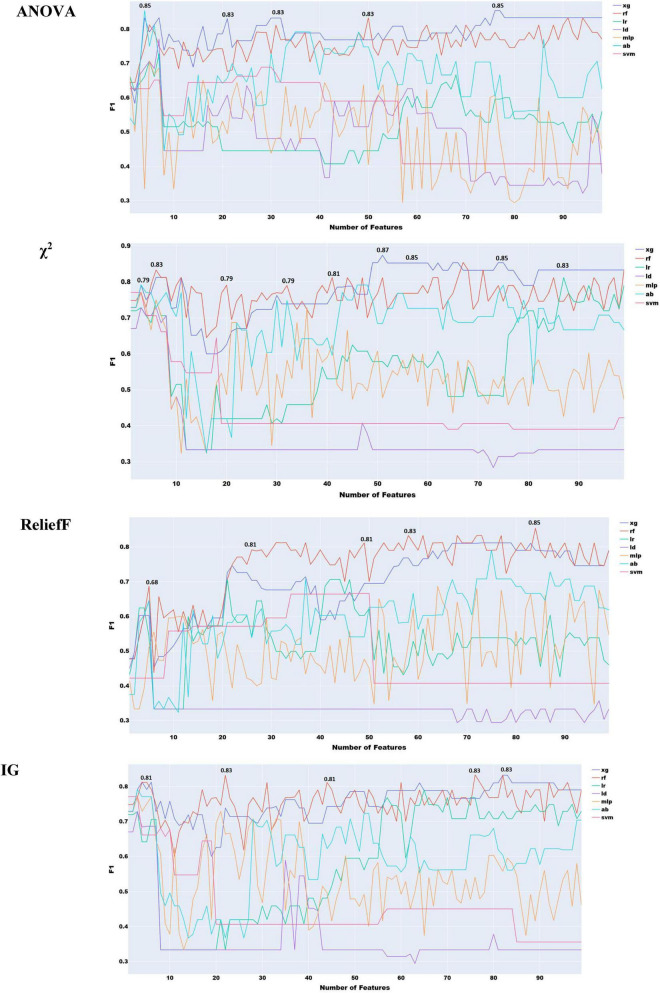
Feature selection experiments with prosodic features only (simple classifiers).

To select the best prosodic features and assess the performance of the classifiers with these features only, we operated in a similar way with the acoustic features. The results of these feature selection methods are presented in Figure 5.

As seen in Figure 5, the best performance of 0.87 with prosodic features was yielded by χ^2^ with XG Boost.

The confusion matrix for the top classifier that produced the best classification performance using 51 prosodic features, χ^2^, and XGBoost is shown in Supplementary Table 22 to identify the error patterns.

### 5.3 Experiments with phonological features

#### 5.3.1 Evaluation of phonological areas

Organizing the features according to phonological domain or category enables a clearer examination of how AD affects each group of features relative to their linguistic functions. Methodologically, although the study does not aim to exhaustively capture every possible phonological feature, it includes a sufficiently broad set to allow the comparison to reflect overall patterns or trends.

**FIGURE 6:**
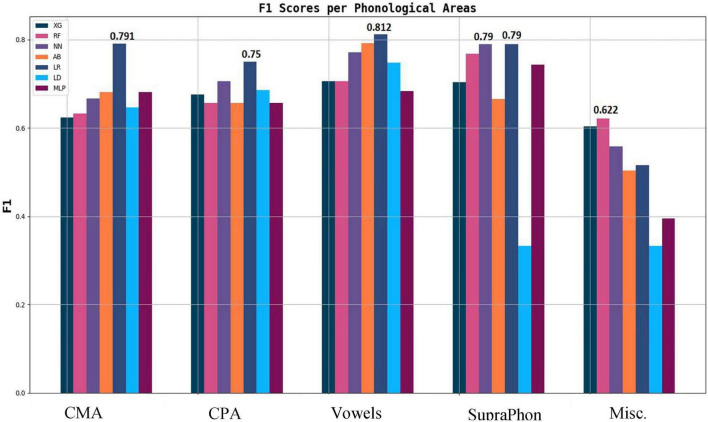
F1 scores of phonological areas.

A few key observations emerge from Figure 6. With the exception of the miscellaneous category, all phonological feature types perform reasonably well. However, their performance remains below the best results obtained using prosodic and acoustic features (Figures 3–5, respectively). Vowel features yield the strongest classification performance, achieving an F1 score of 0.81 with logistic regression, which aligns with their high impact shown in Supplementary Table 18. The number of features within each category does not appear to systematically influence performance. For example, although CMA includes nearly three times as many features as the vowel category, its results are only slightly lower, likely due to higher internal redundancy. Overall, the logistic regression model delivers the best performance across four out of the five linguistic types.

#### 5.3.2 Feature selection experiments

**FIGURE 7:**
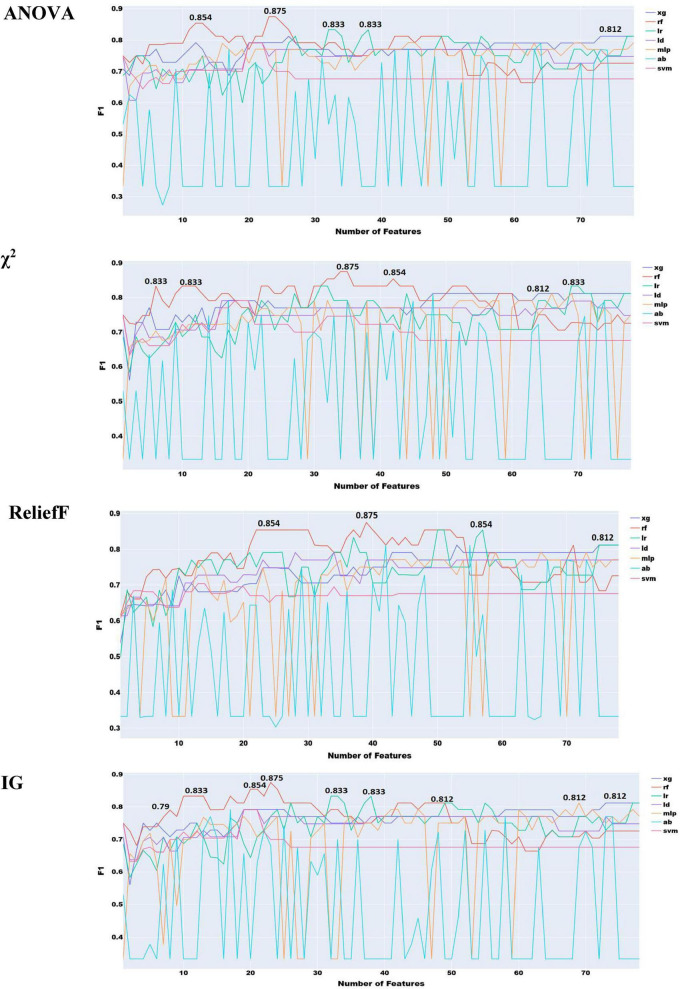
Feature selection experiments with phonological features only (human transcribed).

To identify the optimal subset of features across all linguistic types, we applied the four adopted feature selection methods. Each method ranks features differently, so we constructed a progressively expanding list, starting with the top-ranked feature and adding others incrementally until all features were included (Figure 7). F1 results indicate that adding more features does not always improve classification. This is likely due to internal redundancies. All four methods achieved the same peak F1 of 0.87, with Information Gain and ANOVA reaching optimal performance using fewer features. Despite the small training set, this suggests that phonetic and phonological features alone can yield strong classification performance. This performance is comparable to top performances in the ADReSS Challenge, where models leveraged multiple linguistic levels. This highlights the potential of phonology-based models for early and accurate detection.

Among machine learning algorithms, random forest (RF) consistently performed best across all feature selection methods, followed by XGBoost and logistic regression (LR). These findings underscore the importance of RF and LR, as also observed in the linguistic-type evaluation. In contrast, prior studies focusing on lexical features (e.g., Kurdi, 2024a) did not find a clear algorithmic advantage.

Error patterns are detailed in Supplementary Table 23, which presents confusion matrices for the classifier using 23 ANOVA-selected phonological features with XGBoost. The equal number of false positives and false negatives indicates balanced performance across subgroups.

**FIGURE 8:**
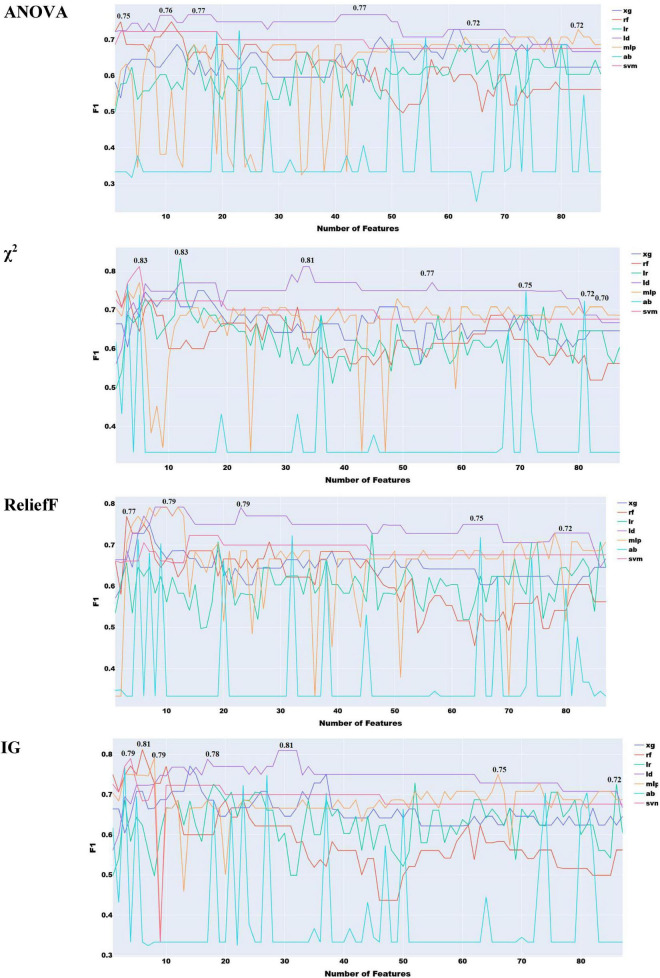
Feature selection experiments with phonological features only (ASR transcribed).

Prior experiments used manually transcribed speech. To assess the impact of automatic speech recognition (ASR), we tested a hybrid approach combining Google and Sphinx modules from Python’s *speech_recognition* library. The Google engine was prioritized because of its better performance, with Sphinx as backup. Results are reported in Figure 8.

As we see in Figure 8, there is an overall decrease of performance across the four feature selection approaches, with χ^2^ giving the best performance of 0.83. As expected, this performance is lower than the best performance with human transcription. This decrease in performance is due to a combination of two factors. ASR modules are still not perfect, and their performance is not yet on par with human hearing (Dhanjal and Singh, 2024; Rista and Kadriu, 2020). The second factor is that the audio files were not collected in ideal condition, making the job of ASR engines harder.

### 5.4 Experiments with prosodic and acoustic features

This section focuses on evaluating the combined use of acoustic and prosodic features. Since both feature types are inherently acoustic, some degree of redundancy is anticipated. Therefore, we do not expect a significant performance gain from their combination. The experiments involving prosodic features follow the same methodology used for other feature types. Initially, the most relevant features from both acoustic and prosodic sets are selected using the mRMR method. Subsequently, all four previously adopted feature selection techniques are applied to the combined feature set.

**FIGURE 9:**
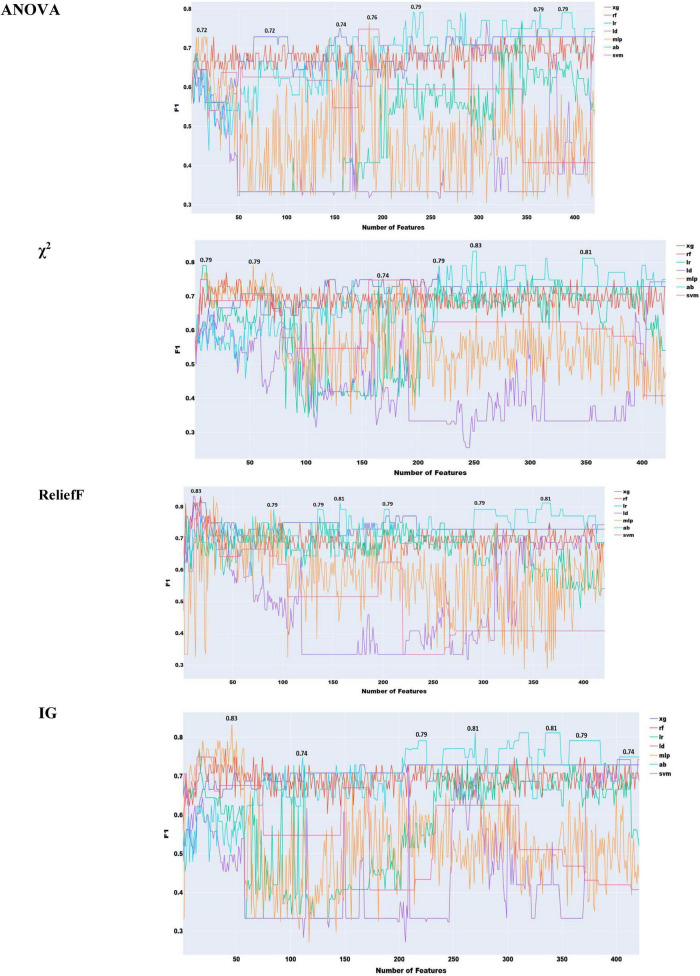
Feature selection experiments with prosodic and acoustic features combined.

As anticipated, combining prosodic and acoustic features did not lead to any performance improvement compared to using them separately, confirming the redundancy between these two feature sets (Figure 9). The highest performance achieved was 0.83, using χ^2^, Information Gain, and ReliefF, which remains significantly lower than the best results obtained with individual feature types.

### 5.5 Experiments with acoustic and phonological features

The objective of this experiment is to explore the potential complementarity between acoustic and phonological features. Unlike acoustic and prosodic features, which overlap as we saw in section 5.4, acoustic and phonological features are distinct. They capture different aspects of speech. Acoustic features primarily capture low-level signal characteristics, while phonological features reflect higher-level linguistic information. Due to this inherent difference, we anticipate a stronger complementary effect between these two groups, which could lead to improved performance. To conduct this analysis, we utilized the combined feature set which resulted in a total of 383 features being used in the experiments.

**FIGURE 10:**
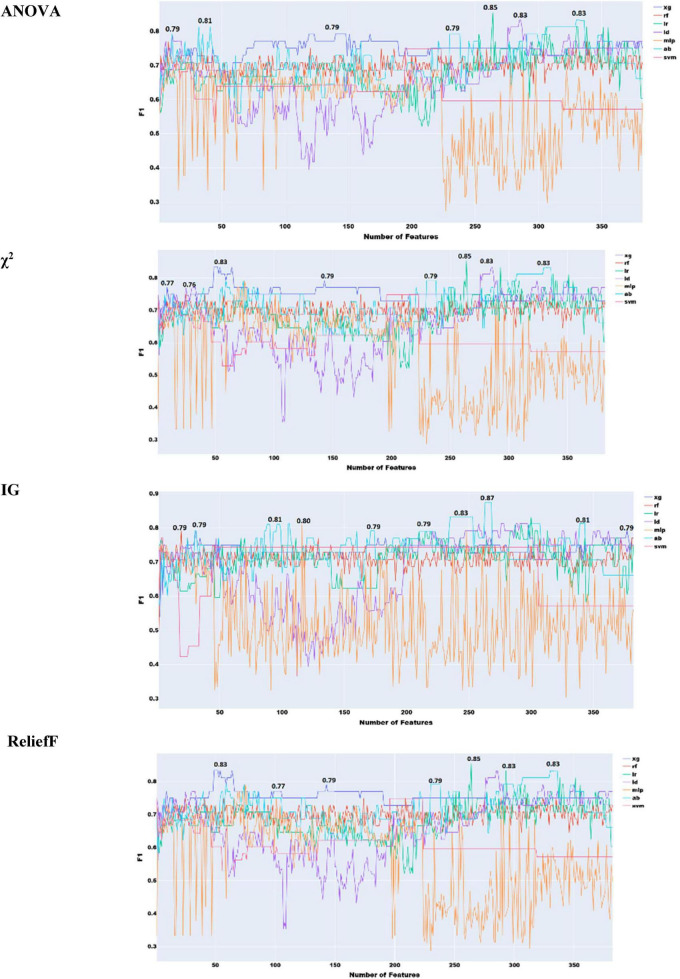
Feature selection experiments with acoustic and phonological features.

As illustrated in Figure 10, the combination of acoustic and phonological features does not lead to any noticeable improvement over the use of either feature set independently. In fact, the highest performance achieved with the combined feature set is 0.87, which is identical to the best result obtained using only phonological features with roughly the same number of features. This outcome indicates that, despite the theoretical differences between acoustic and phonological features, their practical complementarity is limited in this context. The lack of performance gain suggests that the information captured by each set may overlap more than initially expected, or that the current feature selection and modeling approach may not be effectively leveraging the differences between the two.

### 5.6 Experiments with prosodic and phonological features

In this experiment, we investigate the effectiveness of combining two subgroups of features: phonological and prosodic. These feature also types differ fundamentally in nature, phonological features are typically discrete, representing symbolic or categorical information about the sound structure of speech (such as phonemes or syllables), whereas prosodic features are continuous, capturing dynamic aspects of speech such as pitch, F0, and amplitude. Despite their differences, both feature sets relate to speech rhythm and structure, and may offer complementary information. For example, features like speech rate, measured in words per second, syllables per second, or phonemes per second, span across multiple linguistic domains, integrating acoustic, phonological, and even morphological cues.

In total, this combined feature set consists of 153 features, initially obtained using mRMR. The features were then ranked using our four adopted selection methods to determine their relative importance. This ranking helps reveal which features, across both prosodic and phonological domains, contribute most significantly to performance and highlights how cross-domain features such as speech rate and turn length may bridge gaps between linguistic levels.

**FIGURE 11:**
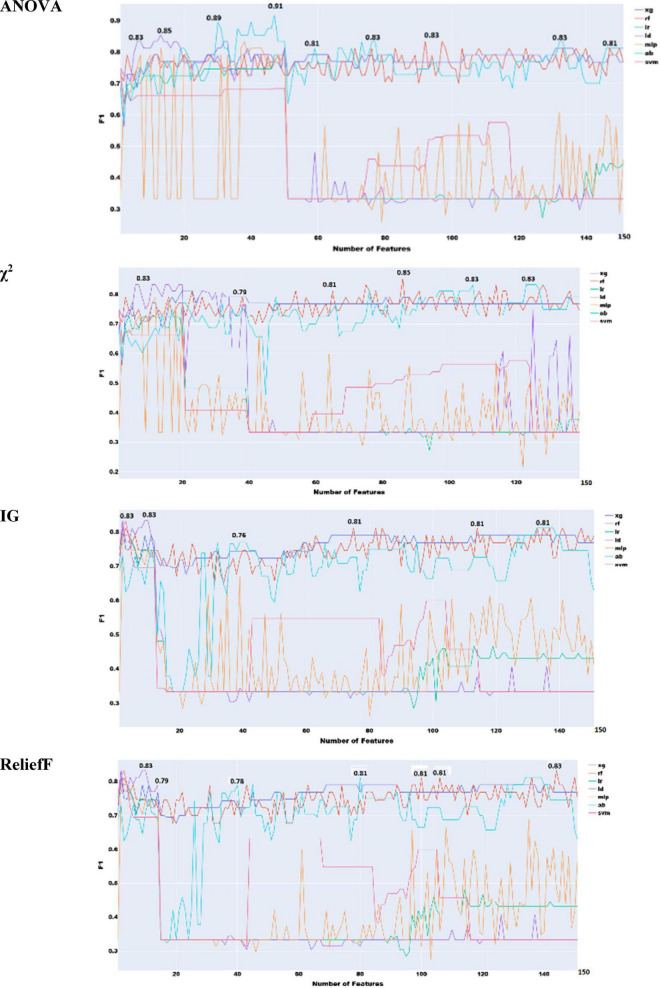
Feature selection experiments with prosodic and phonological features.

As seen in Figure 11, the best overall performance (F1 = 0.87) is achieved using the four feature selection approaches with the combination of prosody and phonology. This performance is the same achieved by prosody and phonology individually. This suggests that combining both types does not lead to improvement.

### 5.7 Experiments using the best of phonological, acoustic, and prosodic features

**FIGURE 12:**
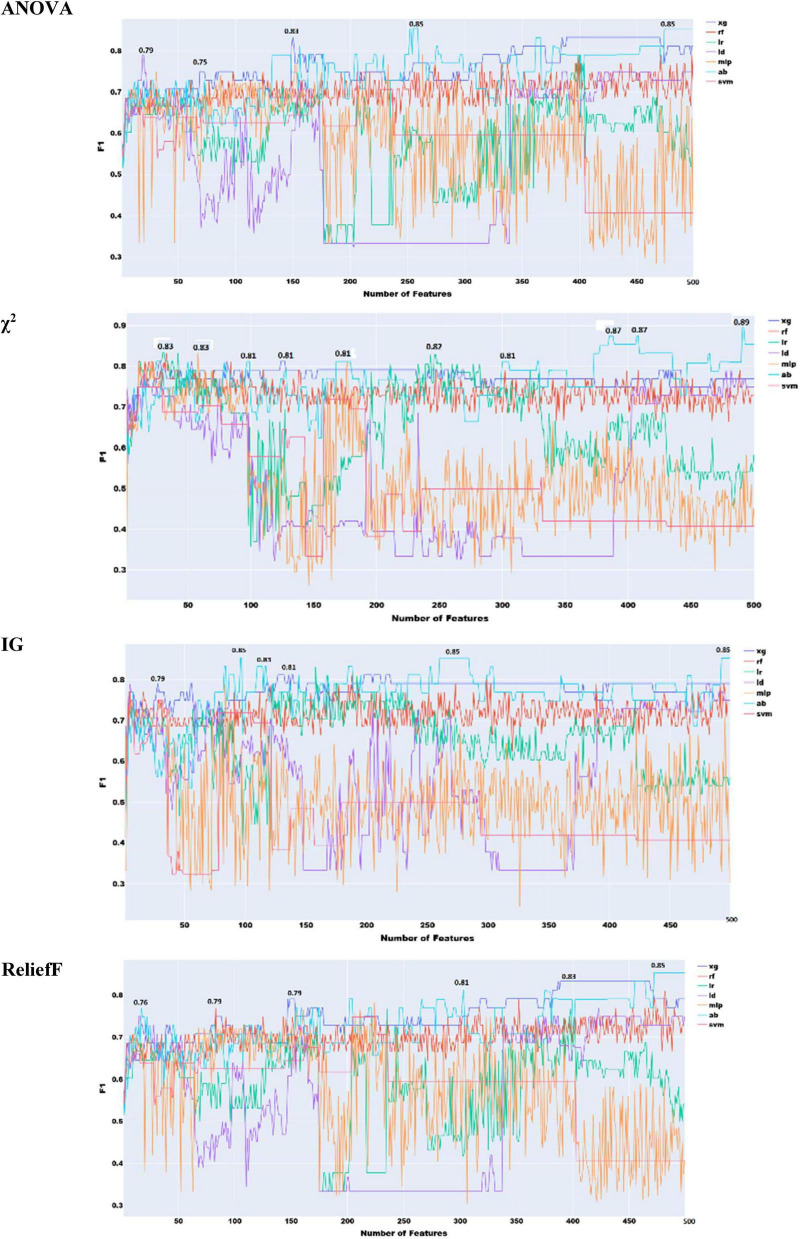
Feature selection experiments with all the speech features combined.

The combination of all the speech features used in the above sections within one set of features can help identify an optimal combination of features that can lead to the best performance. As illustrated in Figure 12, χ^2^ feature selection yielded the highest F1 score (0.89), outperforming all other conventional machine learning approaches. Using logistic regression, this method benefited from the broad coverage of prosodic, acoustic, and phonological features, enabling robust AD detection from speech. This performance was also obtained using χ^2^ and soft voting ensemble learning but with 227 features only (less than the 256 used by the classifier built with acoustic features only). Notably, this performance is equivalent to that of the ensemble model trained on acoustic features alone.

The confusion matrix (Supplementary Table 24) illustrates the top classifier’s performance, which employed soft voting ensemble learning with χ^2^-selected features. The model accurately identified 20 AD cases and 23 non-AD cases, producing only one false positive but missing four AD cases, indicating a conservative bias that minimizes false positives. While this results in slightly lower recall, there is potential to capture more AD cases without compromising specificity.

Although the F1 score is marginally below the 0.91 reported by Kurdi (2024a) using lexical features on the same data set, it remains near state-of-the-art for models relying solely on automatically extracted speech features. These findings emphasize the diagnostic value of acoustic, prosodic, and phonological cues and underscore the importance of careful feature selection in early dementia detection.

### 5.8 Feature ranking

**FIGURE 13:**
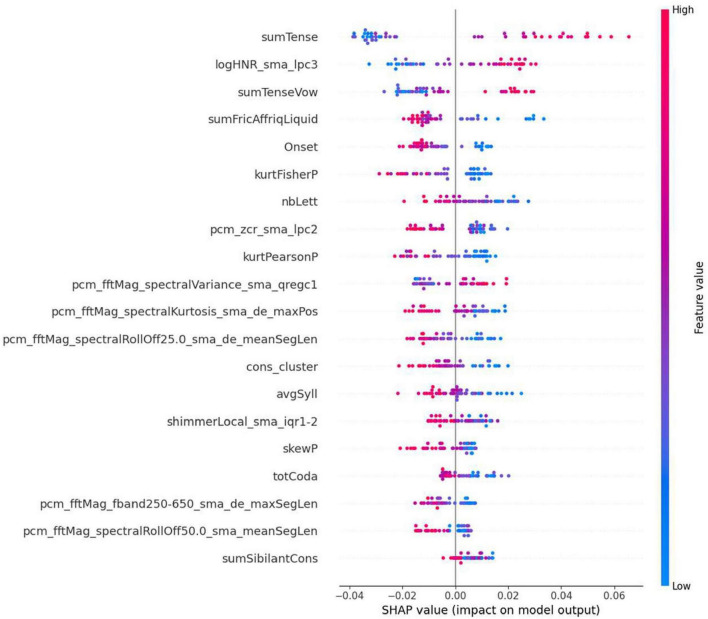
SHAP plot showing the top 20 most important features based on the classifier presented in Figure 12.

To discuss the contribution of individual features that contributed to the top performance shown in Figure 12, a SHapley Additive exPlanations (SHAP) diagram is provided in Figure 13.

The SHAP summary plot (Figure 13) reveals that the top twenty features encompass a diverse mix of phonological (e.g., sumTense and cons_cluster), acoustic (e.g., logHNR_sma_lpc3 and pcm_fftMag_spectralVariance_sma_qreg1), and prosodic measures (e.g., shimmerLocal_sma_iqr1-2). This variety indicates that the model effectively incorporates information from multiple speech domains. The presence of features from different areas, with no single domain dominating the predictions, highlights that the model utilizes complementary cues across speech dimensions. This validates our decision to use a comprehensive feature set, which contributes to the model’s robust overall performance.

The small number of false negatives is notable, but further improvements could be made by expanding training data or incorporating multimodal inputs. Overall, the model shows promise as a non-invasive, speech-based screening tool.

### 5.9 Generalization experiments using the University of Delaware’s MCI dataset

To evaluate the generalizability of our model trained on the ADReSS Challenge dataset, we tested it on the University of Delaware (UD) corpus, which includes speech from individuals with Mild Cognitive Impairment (MCI), a related but less severe neurodegenerative condition. Unlike the ADReSS dataset, which contains only Cookie Theft picture descriptions, the UD corpus spans diverse elicitation tasks, including multiple picture descriptions (Cookie Theft, Cat Rescue, Going and Coming), story narratives (Cinderella), procedural narratives (peanut-butter-and-jelly sandwich), and personal narratives (Hometown). This task heterogeneity poses a substantial test of robustness, reflecting the difficulty of applying a model trained on a single, uniform task to more variable and ecologically complex speech. To maintain comparability with ADReSS, a 70–30 train–test split was used for the UD data.

As shown in Table 1, performance declines when models trained on Cookie Theft AD speech are tested on the more varied UD corpus. This drop underscores the challenge of transferring models across disorders (AD → MCI) and across task types. Because AD typically produces more pronounced impairments than MCI, F1 scores for detecting MCI are consistently lower than for detecting AD, though the results still show that the model captures milder deficits to a meaningful extent. Phonological and acoustic features generalize best, whereas prosodic features perform poorly, likely due to their sensitivity to task structure and individual speaking style.

**TABLE 1:** Results of the generalization tests using the UD corpus.

Linguistic area	Type	Best F1 MCI model	Best F1 AD model
Phonology	Simple	0.69	0.61
Acoustics	Simple	0.67	0.594
Prosody	Simple	0.5	0.39
All	Simple	0.65	0.60
All	Ensemble	0.74	0.64

Combining all feature types improves performance, and the ensemble model yields the strongest results (F1 = 0.74 for MCI; 0.64 for AD), indicating that integrating complementary linguistic and acoustic information enhances robustness.

Overall, these findings show moderate cross-dataset generalizability while underscoring the limitations of training on a narrowly defined elicitation task when broader clinical applicability is desired. The results further confirm that the model trained on the full feature set provides superior performance relative to more restricted feature subsets like the one trained on acoustic features only that has a similar F1 performance of 0.89.

### 5.10 Generalization experiments using the Ronald Regan dataset

The Reagan dataset was used to evaluate how well our AD model, trained on the ADReSS Challenge data, generalizes to a different AD-related corpus. This dataset introduces several notable challenges. First, people with Alzheimer’s may show fluctuating speech patterns, with periods in which cognitive symptoms are less evident. Second, the recordings were produced under inconsistent conditions, leading to differences in audio quality, environment, and microphone setup. Together, these factors add realistic variability that makes the dataset both difficult to model and valuable for assessing speech-based detection of cognitive decline. Because of the limited size of this dataset, only the results using all the features will be presented (Table 2).

**TABLE 2:** Results of the generalization tests using the Regan corpus using all the features.

Best F1 Regan’s data model	Best F1 ADReSS data model
0.77	0.61

The model obtained an F1 score of 0.77 on the Reagan dataset (both training and testing), exceeding its performance on the ADReSS test set (F1 = 0.61). This result suggests that the model generalizes reasonably well to an external corpus despite substantial variability in recording conditions and speech quality. This performance may be attributable to a pronounced phonological, prosodic and acoustic manifestations of cognitive decline in Reagan’s later speeches, which align with the patterns learned from the ADReSS training data.

## 6 Discussion and Limitations

### 6.1 General discussion

To contextualize our findings, we compared our results with prior studies using the ADReSS Challenge dataset, allowing a direct evaluation of speech-based AD detection performance. Luz et al. (2020) used a comprehensive acoustic feature set, including MFCCs, jitter, shimmer, and harmonic-to-noise ratios, achieving an F1 score of 0.62 with speech features alone. Martinc and Pollak (2020) applied clustering to MFCCs and eGeMAPS features with audio duration and linguistic embeddings, reporting 0.77 accuracy. Balagopalan et al. (2020) combined speech and semantic features, reaching F1 = 0.84 with an SVM classifier. Cummins et al. (2020) explored Bag-of-Audio-Words representations and deep learning models, achieving F1 scores between 0.62 and 0.73 depending on features and architecture. Other notable approaches include Pappagari et al. (2020), who leveraged x-vector embeddings from speaker recognition (up to 0.66 accuracy), Edwards et al. (2020), who integrated OpenSMILE, GeMAPS, and ComParE acoustic descriptors with phonemic data (F1 = 0.78), and Sarawgi et al. (2020), who focused on disfluency and low-level descriptors, achieving 0.83 accuracy.

Our findings indicate that ensemble models leveraging acoustic information, together with the model based on the integration of acoustic, prosodic, and phonological features, outperform previously reported speech-only approaches. These findings suggest that a systematic exploration of multiple feature sets and machine learning algorithms can be beneficial for speech-based AD classification. Evaluation on the Delaware corpus confirmed that combining the three types of speech information enhances generalization, as it achieved the best result.

Recent work has emphasized that the clinical value of speech-based digital biomarkers lies not only in classification performance but also in their robustness and longitudinal stability. Spilka et al. (2025) showed that a speech-derived biomarkers from Clinical Dementia Rating (CDR) interviews exhibited moderate test–retest reliability (Interclass Correlation Coefficient (ICC) ≈ 0.62). They also showed that it can help track clinical progression across standard clinical scales like ADAS-Cog, CDR-SB, and ADCS-ADL, despite not reporting traditional machine learning metrics such as F1 or accuracy. This framework underscores the importance of external validity, longitudinal sensitivity, and clinical correlation in biomarker evaluation. In this context, our cross-cohort generalization results provide initial evidence of generalization across different recording conditions and datasets. These findings motivate further investigation of robustness using longitudinal and test-retest designs.

### 6.2 Clinical discussion

These findings suggest potential clinical relevance for research on detection of Alzheimer’s disease. Speech is a natural, accessible, and low-burden biomarker that can be collected unobtrusively in primary care or telehealth settings. Our results on the Delaware MCI corpus are aligned with prior evidence that acoustic, prosodic, and phonological changes emerge early in cognitive decline, often preceding deficits detected by standard neuropsychological assessments (Bidelman et al., 2017; Ding et al., 2024).

Models that integrate multiple speech-feature types performed strongly, reflecting AD’s impact across rhythm, articulation, and spectral characteristics. Capturing this multidimensional profile may enable more sensitive and comprehensive screening tools, complementing traditional cognitive tests and supporting earlier identification of at-risk individuals.

Feature level interpretability may support model transparency and facilitate future clinical adoption. Speech-based assessments are non-invasive, low-cost, and easy to administer, offering opportunities to expand cognitive screening, particularly in underserved or remote populations. By focusing on non-lexical speech features, these approaches may reduce reliance on linguistic content, which is known to be sensitive to language proficiency and educational background. Combined with passive collection via mobile or telephone platforms, these features may support scalable, remote screening within routine clinical workflows and community programs, subject to further validation in real world clinical workflows.

Although our findings are favorable relative to prior ADReSS studies, clinical translation requires validation in larger, longitudinal, and more diverse cohorts. Future research should examine longitudinal trajectories of these speech features and their associations with established AD biomarkers, including neuroimaging, cerebrospinal fluid measures, and neuropsychological performance.

### 6.3 Limitations and future work

A primary limitation of this study is its reliance on the ADReSS Challenge dataset. Although this dataset is balanced and widely used for benchmarking, it remains relatively small, demographically homogeneous, and collected under controlled conditions using a single narrative task (the Cookie Theft picture description). These constraints may limit ecological validity and reduce the generalizability of our findings to more diverse, multilingual, or real-world populations. Future work should therefore validate the proposed approach on larger and more heterogeneous datasets that include multiple narrative tasks, fully spontaneous speech, and variable recording environments.

The dataset’s cross-sectional design further restricts the analysis to binary classification of Alzheimer’s disease (AD) versus healthy controls, preventing investigation of disease progression, early detection among at-risk individuals, or longitudinal patterns of speech decline. Future studies should incorporate longitudinal datasets to monitor speech changes over time and relate them to established biomarkers such as neuroimaging, cerebrospinal fluid measures, and standardized cognitive assessments.

This study focuses exclusively on speech, enabling an in-depth examination of acoustic, prosodic, and phonological features in AD. While this provides valuable insight into spoken-language changes, speech represents only one dimension of the broader linguistic impairments associated with AD. This work is part of an ongoing research program: our previous study analyzed lexical features (Kurdi, 2024a), and forthcoming work will address syntax, morphology, and discourse-level phenomena. Integrating these linguistic layers with speech-based models will support a more comprehensive understanding of language impairment in AD.

Although our datasets lack PET/CSF measures, the convergence between our top features and biomarker-associated patterns reported in prior work strengthens the mechanistic plausibility of speech-based detection. Integrating imaging or fluid biomarkers in future work will be essential to determine whether these feature clusters represent valid surrogate indicators of underlying AD pathology.

Another limitation is that the study includes only AD and cognitively healthy controls. In clinical settings, differential diagnosis involves distinguishing AD from other neurological, psychiatric, or cognitive disorders that also affect language, such as depression, stress, and schizophrenia (Alexopoulou et al., 2025; Ehlen et al., 2023). Expanding datasets to include such conditions would enhance real-world applicability.

Cognitive fluctuations within individuals with AD may also have contributed to variability in the samples. Moreover, the Reagan dataset used for generalization consists of a single speaker with heterogeneous recordings. Although the model showed robust generalization performance, future work should evaluate multi-speaker datasets and incorporate methods to handle intra-individual variability, including repeated measures and longitudinal designs.

This paper treats the onset of AD as a discrete process. Future work should instead consider AD onset as continuous process, using longitudinal data both from clinical sources and from renowned authors who were diagnosed or suspected of having AD, like Iris Murdoch and Agatha Christie, whose writings are publicly available (Le et al., 2011; Garrard et al., 2005).

## 7 Conclusion

In conclusion, this study validates a systematic, feature-integrated approach for diagnosing Alzheimer’s disease (AD) from speech, achieving high predictive performance. An F1 score of 0.89 was attained either by ensemble learning on acoustic features alone or by integrating phonological, prosodic, and acoustic features. Integration of multiple feature types yielded the best results on the Delaware corpus used for validation, supporting the methodological advantage of a structured, comprehensive feature set. Feature importance analysis highlighted phonatory instability and temporal disruption as objective acoustic markers of AD. While promising, these findings are based on a controlled dataset; future work should assess their generalizability using longitudinal and ecologically valid speech samples from diverse aging populations.

Looking ahead, the next steps include integrating syntactic, morphological, and discourse-level features with acoustic and prosodic analysis, validating models on larger, multi-task, longitudinal, and multilingual datasets, and expanding classification beyond binary AD versus healthy controls to include other cognitive and psychiatric conditions. Developing robust methods to account for intra-individual variability due to cognitive fluctuations and linking speech features to biological markers and clinical outcomes will further enhance interpretability and translational potential.

Overall, these results reinforce the promise of a multi-feature speech analysis approach as a scalable, low-cost digital biomarker. The methodology is well suited for remote screening within telehealth platforms, supporting earlier, non-invasive detection and timely intervention for individuals at risk of AD, while laying the foundation for combining validated acoustic features with established lexical markers to accelerate clinical adoption.

## Data Availability

Publicly available datasets were analyzed in this study. This data can be found at: https://talkbank.org/dementia/.

## Footnotes

1
https://orangedatamining.com/

2
https://pypi.org/project/pymrmr/
