# Supplementary material for: Integrating acoustic, prosodic, and phonological features for automatic Alzheimer’s detection
Source: Front Aging Neurosci. 2026 Apr 16;18:1786269. doi: 10.3389/fnagi.2026.1786269 (PMC13158574; doi:10.3389/fnagi.2026.1786269)
Supplement: Supplementary file 1 [file Data_Sheet_1.pdf]

Supplementary **Tables**

Type of Feature	Number of Variants	Type of Feature	Number of Variants
Audio spectral	2800	Log Harmonic to Noise Ratio (HNR)	78
MFCC	1400	Voicing Final Unclipped	78
Pulse Code Modulation (PCM) Fast Fourier Transform (FFT)	1700		

Supplementary **Table 1** Key types and counts of the acoustic features

Feat	IG	Anova	Chi	Relief
audSpec_Rfilt_sma_de[21]_meanSegLen	A	A	A	A
audSpec_Rfilt_sma[19]_maxSegLen	A	A	A	B
audSpec_Rfilt_sma[3]_maxPos	A	B	A	B
audSpec_Rfilt_sma_de[20]_maxSegLen	A	A	A	B
audSpec_Rfilt_sma_de[23]_meanSegLen	A	A	A	B
audSpec_Rfilt_sma_de[17]_maxSegLen	A	A	A	C
audSpec_Rfilt_sma_de[18]_maxSegLen	A	A	A	C
audSpec_Rfilt_sma_de[21]_segLenStddev	A	A	A	C
audSpec_Rfilt_sma[20]_maxSegLen	A	A	B	B
audSpec_Rfilt_sma[2]_centroid	A	B	A	C

Supplementary **Table 2** The top 10 audio spectral features

Feat	IG	Anova	Chi	Relief
mfcc_sma[4]_maxSegLen	B	A	B	A
mfcc_sma[4]_segLenStddev	B	B	B	B
mfcc_sma[14]_maxSegLen	A	B	B	B
mfcc_sma[13]_maxSegLen	B	B	B	B
mfcc_sma[10]_maxSegLen	B	B	B	C
mfcc_sma_de[1]_meanSegLen	C	B	C	A
mfcc_sma[13]_segLenStddev	C	B	B	C
mfcc_sma[3]_maxSegLen	C	B	C	B
mfcc_sma[6]_maxSegLen	C	B	C	C
mfcc_sma[7]_range	C	D	C	B

Supplementary **Table 3** The top 10 MFCC features

Feat	IG	Anova	Chi	Relief
pcm_fftMag_spectralKurtosis_sma_de_maxSegLen	B	A	B	A
pcm_fftMag_spectralKurtosis_sma_maxSegLen	A	B	A	A
pcm_fftMag_spectralRollOff50.0_sma_peakRangeAbs	B	C	B	B
pcm_fftMag_spectralSkewness_sma_de_maxSegLen	B	B	B	B
pcm_fftMag_fband1000-4000_sma_de_meanSegLen	B	B	A	C
pcm_fftMag_spectralKurtosis_sma_de_segLenStddev	C	B	B	A
pcm_fftMag_spectralHarmonicity_sma_maxSegLen	A	B	A	D
pcm_fftMag_spectralHarmonicity_sma_meanSegLen	A	B	A	D
pcm_fftMag_fband1000-4000_sma_de_maxSegLen	B	A	B	D
pcm_fftMag_fband1000-4000_sma_maxSegLen	C	A	B	C

Supplementary **Table 4** Top 10 PCM features

Feature	IG	Anova	χ^2	ReliefF
logHNR_sma_lpc3	B	A	B	D
voicingFinalUnclipped_sma_de_kurtosis	D	B	D	C
voicingFinalUnclipped_sma_qregc2	None	None	D	None
logHNR_sma_de_kurtosis	D	D	D	None
voicingFinalUnclipped_sma_de_leftctime	None	None	None	D
logHNR_sma_de_leftctime	D	None	None	None

Supplementary **Table 5** The top 6 LogHNR and voice final unclipped features

Type of feature	Number of Variants	Library
F0 variation measures	39	openSMILE
Pitch	8	Parselmouth & librosa
Amplitude	8	Scipy
Intonation Peaks	9	librosa
Speech rate	3	CMU dictionary & wave
Silence	5	pydub
Jitter	156	openSMILE
Shimmer Local	78	openSMILE
Jitter Local	78	openSMILE
Jitter Differential Dynamic Programming	78	openSMILE
Articulation rate and unfilled pause	8	-

Supplementary **Table 6** Prosodic features extracted from speech files

Feature	IG	Anova	χ^2	ReliefF
F0final_sma_lpc2[1]	A	A	A	D
F0final_sma_maxPos	B	A	A	B
F0final_sma_lpc1	A	B	B	C
F0final_sma_lpc3	B	A	A	None
F0final_sma_leftctime	B	A	B	None
F0final_sma_iqr2-3	B	B	B	C
F0final_sma_qregc3	B	D	A	D
F0final_sma_minPos	B	D	D	None
F0final_sma_quartile3	C	B	B	D
F0final_sma_risetime	B	None	D	D

Supplementary **Table 7** Top 10 F0 features

Feature	IG	Anova	χ^2	ReliefF
meanP	A	A	A	A
pearModeFirstP	A	A	A	A
iqrP	A	A	A	B
skewP	A	A	A	D
kurtFisherP	A	B	A	D
kurtPearsonP	A	B	A	D
stdP	B	D	B	A
varP	B	D	B	B
meanPitchLibroP	B	None	D	D
maxPitchLibroP	C	D	D	D

Supplementary **Table 8** Top 10 pitch features

[1] LPC stands for Linear Predictive Coding, which is a method for representing the digital signal of speech in a compressed form.

Feature	IG	Anova	χ^2	ReliefF
IQR	B	A	B	B
Kurt Pearson	D	C	B	B
mean	A	D	D	D
Pearson Mode First	A	D	D	D
Kurt Fisher	D	C	C	C
max	D	D	None	A
std	None	D	None	A
range	D	None	None	A
min	None	None	None	A
var	None	D	None	B
mode	D	D	None	D
delta	D	None	D	D
Pearson Mode Second	D	D	None	D

Supplementary **Table 9** Top 10 amplitude features

**Feature**	**IG**	**Anova**	**$\chi 2$**	**ReliefF**
Ratio Peaks	A	A	A	A
Mode	C	D	B	D
IQR	B	D	None	B
Range	B	D	D	D
Pearson Mode Second	B	D	D	D
Max	D	D	None	B
Kurt Fisher	None	D	None	B
Kurt Pearson	None	D	None	B
Standard Deviation	D	None	None	C
Delta	D	D	D	None
Variation	D	None	None	D
Pearson Mode First	D	None	None	D
Mean	None	None	None	D

Supplementary **Table 10** Ranking of the intonation peak features

Feature	IG	Anova	χ^2	ReliefF
ratioWords	D	D	D	None
ratioChars	D	D	D	None
ratioSylls	None	D	D	None

Supplementary **Table 11** Ranking of the three articulation rate features

Feature	IG	Anova	χ^2	ReliefF
Ms10	C	D	B	B
Ms200	B	D	C	None
Ms25	D	D	D	D
Ms50	D	D	D	D
Ms100	D	None	None	D

Supplementary **Table 12** Ranking of the unfilled features

Feat	IG	Anova	χ^2	ReliefF
shimmerLocal_sma_de_lpc1	A	A	A	D
shimmerLocal_sma_range	A	B	A	D
shimmerLocal_sma_iqr1-2	B	B	A	D
shimmerLocal_sma_de_kurtosis	C	B	D	B
shimmerLocal_sma_qregerrQ	D	D	C	B
shimmerLocal_sma_de_lpc4	None	B	B	D
shimmerLocal_sma_de_qregerrQ	B	D	D	D
shimmerLocal_sma_de_percentile1	D	B	D	D
shimmerLocal_sma_de_lpc3	None	D	D	B
shimmerLocal_sma_de_quartile2	None	D	D	D

Supplementary **Table 13** Top 10 shimmer features

Feat	IG	Anova	χ^2	ReliefF
jitterDDP_sma_de_kurtosis	B	A	B	B
jitterDDP_sma_range	C	A	B	B
jitterLocal_sma_range	C	A	B	C
jitterLocal_sma_minPos	D	B	B	D
jitterLocal_sma_de_posamean	None	D	D	B
jitterDDP_sma_de_upleveltime90	None	D	D	B
jitterDDP_sma_qregc1	None	D	B	D
jitterDDP_sma_de_qregerrQ	D	D	D	D
jitterDDP_sma_pctlrange0-1	None	D	D	D
jitterDDP_sma_qregc3	None	D	D	None

Supplementary **Table 14** Top 10 jitter features

Type	Nb Features
Consonant Place of Articulation	22
Consonant Manner of Articulation	27
Vowel features	11
Supra-phonemic Structure	11
Misc. features	6

Supplementary **Table 15** Type of phonetic and phonological features

Type	Phonemes	Anova	χ^2	reliefF	IG
Bilabials	p, b, m, w	D	D	-	D
Bilabials to cons		D	-	-	D
Labio-dental	f, v	C	C	C	C
Labio-dental to cons		B	B	C	B
Dental	θ, ð	B	A	B	B
Dental to cons		B	A	A	A
Alveolar	t, d, s, z, n, l	C	C	C	C
Alveolar to cons		C	C	C	C
post-alveolar	ʒ, ʃ	C	-	-	C
post-alveolar to cons		A	-	D	C
Palatal	ʒ, ʃ, tʒ, tʃ, r, y	C	C	D	C
Palatal to cons		C	B	C	C
Glottal	ʔ h	D	D	C	D
Glottal to cons		C	D	C	C
Lateral	l	-	D	D	-
Lateral to cons		-	D	-	-
Coronal	r, l, t, d, ð, θ, n, s, z, ʃ	D	C	-	D
Coronal to cons		C	C	-	C

Supplementary **Table 16** CPA features with their rankings[2] according to four feature ranking methods: Anova, χ^2, reliefF, and Information Gain

Type	Phonemes	Anova	χ^2	reliefF	IG
Stop	p, b, t, d, k, g, ʔ	-	-	-	-
Stop to cons.		-	-	-	-
Fricatives	v, f, θ, ð, z, s, ʒ, ʃ, h, tʒ, tʃ	B	B	A	B
Fricatives to cons		A	A	A	A
FriAffLiq	v, f, θ, ð, z, s, ʒ, ʃ, t, d, l, r, h	A	B	C	A
FriAffLiq to cons		A	A	A	A
Nasal	m, n, ŋ	D	C	-	D
Nasal to cons.		-	D	D	-
Liquids	l, r	D	C	-	D
Liquids to cons.		D	C	-	D
Glide	w, y	C	B	A	B
Glide to cons.		B	C	A	B
Approximant	w, r, j	C	-	D	C
Approximant to cons.		-	D	D	-
Sonorant	y, w, l, r, m, n, ŋ	-	D	D	-
Sonorant to cons.		-	-	-	-
Continuant	r, l, θ, ð, n, s, ʃ, k, h	D	-	B	D
Continuant to cons		-	-	C	-
Voiced	b, d, g, v, ð, z, ʒ, ʤ, m, n, ŋ, l, r, ɹ, w, j	D	D	D	D
Voiced to cons.		-	D	D	-
Strident	f, v, s, z, ʃ, ʒ, tʃ, dʒ	B	C	D	B
Sibilant	s, z, ʃ, ʒ, tʃ, dʒ	C	C	D	C
Sibilant to cons.		B	B	D	B
Distributed	s, z	C	C	D	C
Distributed to cons		C	C	C	C
High	w, ʃ, k, g	-	-	B	-
High to cons		-	-	C	-

Supplementary **Table 17** Ranking of CMA features according to the four adopted feature selection methods

Type	Phonemes	Anova	χ^2	reliefF	IG
High to phon	i, ɪ, u	D	C	C	D
High to vow		C	C	C	C
Low to phon	æ, ʌ, a	-	D	-	-
High to vow		D	D	C	D
Round to phon	u, w	C	C	C	C
Round to vow		B	B	B	B
Tense to phon	i, u, e, æ	A	A	C	A
Tense to vow		A	A	C	A
Syllabic to phon	i, e, u, ɑ, ɪ	B	B	C	B
Syllabic to vow		A	A	A	A

Supplementary **Table 18** Rankings of the vowels features according to the four adopted feature selection techniques

Feature	Sounds	Anova	χ^2	reliefF	IG
Syllabic cons. to phon.	m, n, l	B	B	C	B
Syllabic cons. to cons.		B	B	B	B
Consonantal	r, l, p, t, d, ð, θ, n, s, z, ʃ, k, h	D	D	D	D
Diphtongues to phon.	oʊ, aʊ, aɪ, eɪ, ɔɪ, ɪər, ɛər, ʊər	-	-	B	-
Diphtongues to cons.		D	-	-	D

Supplementary **Table 19** Rankings of Misc. phonemic features according to the four adopted feature selection techniques

Type	Anova	χ^2	reliefF	IG
avgSyll	A	A	A	A
consCluster	A	A	A	A
endConson	-	D	B	-
lenOnset	C	C	C	C
lenCoda	B	B	B	B
primStress	D	D	C	D
secondStress	D	D	D	D
frontSyll	D	D	-	D
backSyll	-	-	D	-
diphSyll	D	D	-	D

Supplementary **Table 20** Rankings of the supra phonemic features according to the four adopted features selection methods

Top classifier	F1	TN	FP
Feature Selection: Information gain<br>Number of features: 256<br>**ML algorithm:** adaptive boosting	0.85	20	4
		FN	TP
		3	21

Supplementary **Table 21** Confusion matrix of the top classifier with acoustic features

**Top classifiers**	**F1**	**TN**	**FP**
Feature Selection: χ^2<br>Number of features: 51<br>ML algorithm: XGBoost	0.87	TN	FP
		19	5
		FN	TP
		3	21

Supplementary **Table 22** Confusion matrices of the top classifier with prosodic features

Top classifiers	F1	TN	FP
Feature Selection: Anova<br>Number of features: 23<br>ML algorithm: XGBoost	0.87	21	3
		FN	TP
		3	21

Supplementary **Table 23** Confusion matrix of one of the top classifiers with phonological features

**Top classifier**	**F1**	**TN**	**FP**
Feature Selection: χ^2<br>Number of features: 227<br>**ML algorithm:** soft voting	0.89	23	1
		FN	TP
		4	20

Supplementary **Table 24** Confusion matrix of the top classifier with all the speech features

Supplementary **Figures**

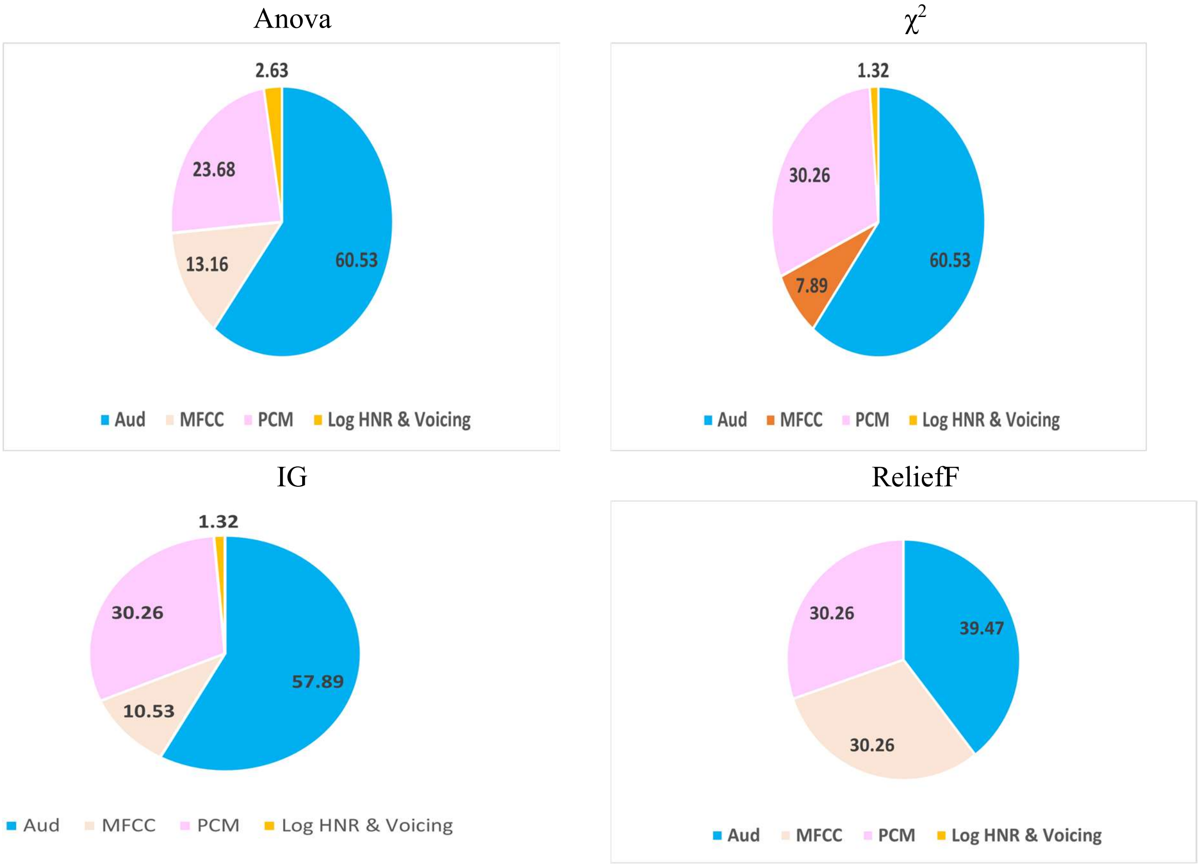

Supplementary **Figure 1** Top 25% of the Acoustic features according to the five feature ranking methods

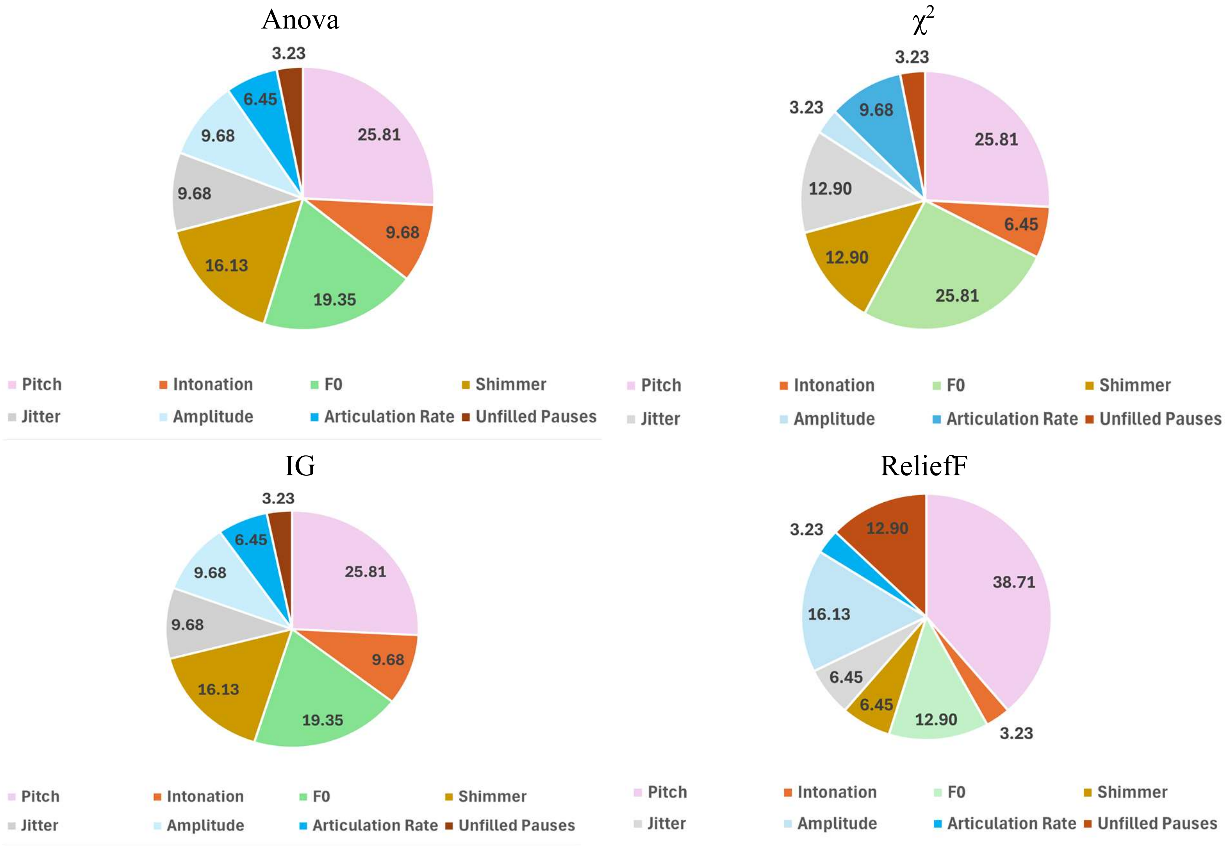

Supplementary **Figure 2** Top 25% of the Prosodic features according to the five Feature Ranking Methods

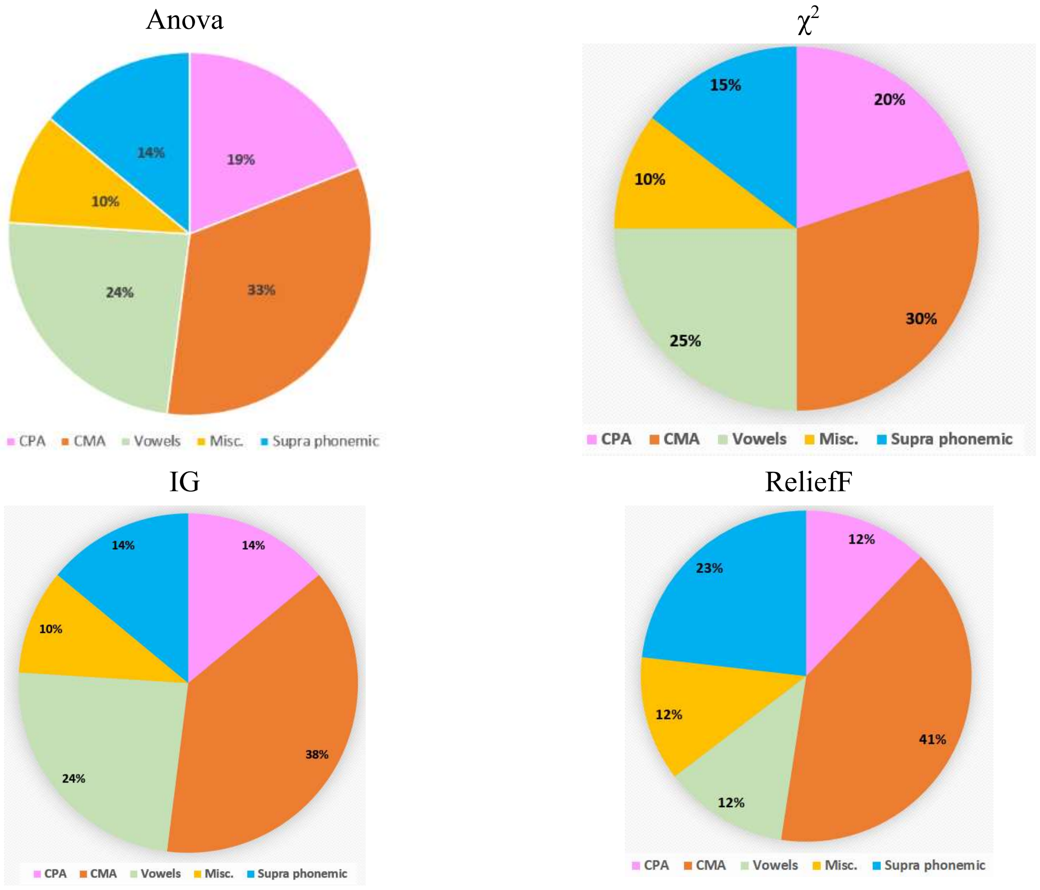

Supplementary **Figure 3** Partition of the top 25% of the phonological features according to the four adopted feature ranking methods
